# N7-Methylation of the Coronavirus RNA Cap Is Required for Maximal Virulence by Preventing Innate Immune Recognition

**DOI:** 10.1128/mbio.03662-21

**Published:** 2022-01-25

**Authors:** Ruangang Pan, Eveline Kindler, Liu Cao, Yu Zhou, Zhen Zhang, Qianyun Liu, Nadine Ebert, Roland Züst, Ying Sun, Alexander E. Gorbalenya, Stanley Perlman, Volker Thiel, Yu Chen, Deyin Guo

**Affiliations:** a State Key Laboratory of Virology, Modern Virology Research Center, Institute for Vaccine Research and RNA Institute, College of Life Sciences, Wuhan University, Wuhan, People’s Republic of China; b School of Medicine, Sun Yat-sen University, Guangzhou, People’s Republic of China; c Institute for Virology and Immunology IVI, Mittelhäusern, Switzerland; d Department of Infectious Diseases and Pathobiology, Vetsuisse Facility, University of Bern, Bern, Switzerland; e Department of Microbiology and Immunology, University of Iowa, Iowa City, Iowa, USA; f Department of Medical Microbiology, Leiden University Medical Center, Leiden, The Netherlands; g Faculty of Bioengineering & Bioinformatics, Lomonosov Moscow State University, Moscow, Russia; h School of Mathematics and Statistics, Wuhan University, Wuhan, China; i Spiez Laboratory, Federal Office for Civil Protection, Spiez, Switzerland; The Pirbright Institute; St. Jude Children's Research Hospital

**Keywords:** coronavirus, nonstructural protein 14 (nsp14), RNA cap structure, N7-methylation, type I interferon, SARS-CoV-2, RNA methylation, immune response, interferons

## Abstract

The ongoing coronavirus (CoV) disease 2019 (COVID-19) pandemic caused by infection with severe acute respiratory syndrome CoV 2 (SARS-CoV-2) is associated with substantial morbidity and mortality. Understanding the immunological and pathological processes of coronavirus diseases is crucial for the rational design of effective vaccines and therapies for COVID-19. Previous studies showed that 2′-O-methylation of the viral RNA cap structure is required to prevent the recognition of viral RNAs by intracellular innate sensors. Here, we demonstrate that the guanine N7-methylation of the 5′ cap mediated by coronavirus nonstructural protein 14 (nsp14) contributes to viral evasion of the type I interferon (IFN-I)-mediated immune response and pathogenesis in mice. A Y414A substitution in nsp14 of the coronavirus mouse hepatitis virus (MHV) significantly decreased N7-methyltransferase activity and reduced guanine N7-methylation of the 5′ cap *in vitro*. Infection of myeloid cells with recombinant MHV harboring the nsp14-Y414A mutation (rMHV_nsp14-Y414A_) resulted in upregulated expression of IFN-I and ISG15 mainly via MDA5 signaling and in reduced viral replication compared to that of wild-type rMHV. rMHV_nsp14-Y414A_ replicated to lower titers in livers and brains and exhibited an attenuated phenotype in mice. This attenuated phenotype was IFN-I dependent because the virulence of the rMHV_nsp14-Y414A_ mutant was restored in *Ifnar*^−/−^ mice. We further found that the comparable mutation (Y420A) in SARS-CoV-2 nsp14 (rSARS-CoV-2_nsp14-Y420A_) also significantly decreased N7-methyltransferase activity *in vitro*, and the mutant virus was attenuated in K18-human ACE2 transgenic mice. Moreover, infection with rSARS-CoV-2_nsp14-Y420A_ conferred complete protection against subsequent and otherwise lethal SARS-CoV-2 infection in mice, indicating the vaccine potential of this mutant.

## INTRODUCTION

The ongoing coronavirus (CoV) disease 2019 (COVID-19) pandemic caused by severe acute respiratory syndrome CoV 2 (SARS-CoV-2) remains a major threat to human health. CoVs encode several genes that are able to antagonize responses of the innate immune system, contributing to virulence. The host immune system senses viral constituents, such as uncapped viral RNA or replication intermediates, initiating and propagating the initial antiviral signaling cascade after infection (1, 2). The 5′ end of eukaryotic mRNAs possesses a cap structure, which is required for pre-mRNA splicing, mRNA export, RNA stability, and translational initiation. *S*-Adenosyl methionine (SAM)-dependent (guanine) N7-methyltransferase (N7-MTase) methylates eukaryotic mRNAs at the N7 position of the cap guanylate to produce a cap-0 structure (m7GpppN) (3). This minimal cap structure is further modified in higher eukaryotes by methylation at the ribose 2′-O position of the first nucleotide by a SAM-dependent ribose 2′-O-methyltransferase (2′-O-MTase), forming a cap-1 (m7GpppNm) structure (3, 4). In the absence of 2′-O-methylation, RNA activates the innate immune response (5, 6). As a consequence, most eukaryotic viruses have evolved strategies for capping their RNAs by both N7- and 2′-O-methylation to mimic host mRNAs.

The antiviral immune response is initiated by a variety of pathogen-associated molecular patterns (PAMPs) binding to pattern recognition receptors (PRRs) that are expressed in the endosomes (e.g., Toll-like receptors [TLRs]) or in the cytosol (e.g., retinoic acid-inducible gene I [RIG-I]-like receptors [RLRs]) (7). To distinguish host cell-derived mRNAs from viral RNAs, PRRs have evolved to specifically recognize self and non-self RNAs. The engineered absence of 2′-O-methylation in viral RNA resulted in initiation of the type I interferon (IFN-I) response via signaling via an RLR, MDA5 (melanoma differentiation-associated protein 5), indicating that 2′-O-methylation of RNAs provided a molecular signature to distinguish self and non-self RNAs (5, 6). Viruses of the Alpha- or Sindbis-like supergroup, such as chikungunya virus, Sindbis virus, Semliki forest virus, and hepatitis E virus, possess only an N7-methylated RNA (cap-0) (8–10). Given that these viruses evade the IFN-I response, N7-methylation may be expected to counter innate immune recognition and enhance viral pathogenicity, independently of its effects on translation.

Experimentally characterized CoVs belong to the subfamily *Orthocoronavirinae* in the family *Coronaviridae* of positive-sense single-stranded RNA viruses with a broad vertebrate host range, including humans (11). They include three deadly human pathogens, SARS-CoV-2 and SARS-CoV of the species *Severe acute respiratory syndrome-related coronavirus* and Middle East respiratory syndrome coronavirus (MERS-CoV) of the species *Middle East respiratory syndrome-related coronavirus* in the genus *Betacoronavirus* (12), and cause respiratory disease with substantial mortality (13–17). CoVs, like other RNA viruses, evade the innate immune response by methylating GpppN-RNA, using a virus-encoded SAM-dependent N7-MTase (nonstructural protein 14 [nsp14]) and a SAM-dependent 2′-O-MTase (nsp16) (18–21). The structure and function of coronaviral nsp14 are highly conserved, including those of SARS-CoV, MERS-CoV, SARS-CoV-2, and mouse hepatitis virus (MHV). The N7-MTase core domain is located at the C terminus of nsp14, while a 3′-to-5′ exoribonuclease (ExoN), a protein required for fidelity of genomic replication, resides in the N-terminal half of the same protein (18, 21–25).

MHV is classified within the species *Murine coronavirus* of the genus *Betacoronavirus*, which also includes the human coronaviruses (HCoVs) HCoV-OC43 and HCoV-HKU-1, members of two other species (26–28). MHV infects the livers, intestines, and brains of mice, causing acute hepatitis, enteritis, encephalitis, and a subacute/chronic demyelinating disease dependent upon virus strain (29). Here, we show that N7-methylation is necessary to prevent MDA5-mediated recognition of viral RNA in cells and mice infected with MHV, using mutants with diminished N7-MTase activity and impaired formation of the viral RNA cap-0 structure. The pathogenicity of mutant MHV lacking N7-methyltransferase decreased in wild-type (WT) but not *Ifnar*^−/−^ mice. The comparable recombinant SARS-CoV-2 mutant (rSARS-CoV-2_nsp14-Y420A_) deficient in N7-methyltransferase activity was also attenuated in infected mice. Our results demonstrate an important role for the coronavirus guanine N7-methyltransferase in pathogenesis.

## RESULTS

### Identification of residues critical for N7-MTase function.

**FIG 1:**
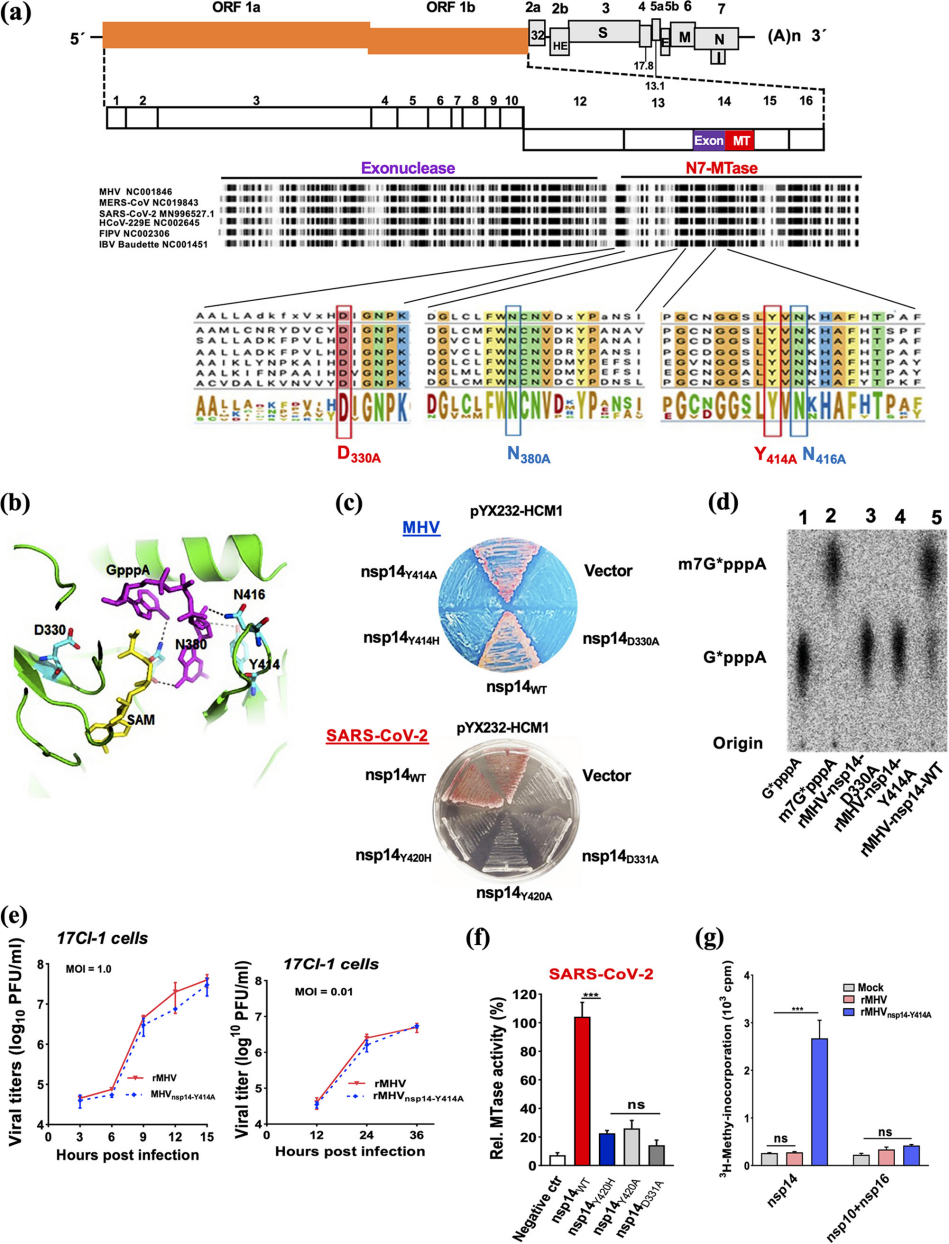
Sequence alignment of CoV-nsp14 and assessment of N7-MTase activity. (a) MHV-A59 genome organization and sequence alignment of the N7-MTase domain of nsp14 from several CoVs. The complete protein sequence of several CoV nsp14s was obtained from GenBank for representative α, β, and γ CoVs. The putative exonuclease (N terminus) and N7-methyltransferase (N7-MTase) (C terminus) domains are shown. Conserved residues within the N7-MTase are highlighted in red and blue, and their numbering corresponds to the location within MHV nsp14. MHV, mouse hepatitis virus strain A59; SARS-CoV, severe acute respiratory syndrome CoV; MERS-CoV, Middle East respiratory syndrome CoV; SARS-CoV-2, severe acute respiratory syndrome CoV 2; HCoV-229E, human coronavirus 229E; FIPV, feline infectious peritonitis virus; IBV, infectious bronchitis virus. The GenBank accession number is shown for each sequence. (b) Structure of MHV nsp14 with the substrates GpppA (in magenta) and SAM (in yellow) and residues (D330, N380, Y414, and N416) as indicated. MHV nsp14 is modeled based on SARS-CoV nsp14 (PDB accession no. 5C8S). Cyan, carbon; red, oxygen; blue, nitrogen. (c) Yeast complementation assay to examine the RNA cap guanosine-N7-methylation function of MHV and SARS-CoV-2 nsp14 mutants, as indicated. YBS40 was complemented by WT MHV nsp14 and the positive control, pYX232-HCM1, but not by MHV nsp14 mutants (with the mutation Y414A or Y414H, with D330A as a negative control). (d) TLC analysis of P1-resistant cap structures released from G*pppA-RNA (substrate) treated with bacterium-derived MHV nsp14-D330A, MHV nsp14-Y414A, and WT MHV nsp14. The positions of origin and migrations of m7G*pppA and G*pppA are indicated on the left (* indicates ^32^P labeled). (e) Infection of murine 17Cl-1 cells with rMHV or rMHV_nsp14-Y414A_ at an MOI of 1 (left) or 0.001 (right). The titer of the virus was determined at the indicated time points. (f) GpppA-RNA was used to test the methylation activities of SARS-CoV-2 nsp14 and its mutants, as indicated. ctr, control. (g) *In vitro* N7-methylation assay using bacterially expressed MHV nsp14 or MHV nsp10 and MHV nsp16. Total poly(A)-containing RNAs were isolated from the culture supernatants of neuro-2A cells infected with rMHV and rMHV_nsp14-Y414A_. Incorporation of ^3^H was measured after treatment with the indicated proteins in the presence of ^3^H-labeled SAM. ns, not significant; ***, *P < *0.001 (unpaired Student *t* test). Data are representative of three independent experiments (mean values ± SD).

We previously identified the residues essential for MTase activity and SAM binding of nsp14 (24). To explore the role of nsp14 N7-MTase activity in virus replication, pathogenicity, and the innate immune response, we selected four highly conserved residues of nsp14 for mutagenesis using comparative sequence analysis of several CoVs, including SARS-CoV-2, and two distantly related invertebrate nidoviruses, roniviruses and mesoniviruses (30). These residues were D331/330, N386/380, Y420/414, and N422/416 (SARS-CoV-2/MHV nomenclatures). All of the selected nsp14 residues were invariant in CoVs, including SARS-CoV-2 (Fig. 1a), but varied to different degrees in the invertebrate nidoviruses. These residues were tentatively assigned to beta-strand structure elements of the core of the MTase fold, with D331/330 implicated in SAM binding and N386/380, Y420/414, and N422/416 located in close proximity to, and implicated in interactions with, the guanine and ribose moieties of GpppA (Fig. 1b) (31). These findings were confirmed and elaborated upon when the structures of SARS-CoV and SARS-CoV-2 nsp14 were solved (31, 32).

Based on these results, we engineered a set of viruses mutated in each of these residues, based on an MHV background. We readily recovered a recombinant MHV nsp14-D330A mutant (rMHV_nsp14-D330A_) that displayed exactly the same phenotype as described previously (33). rMHV_nsp14-D330A_ or the recombinant nsp14-D330A protein/gene is therefore used here in some assays as a control but is not described in further detail. Instead, we focused on MHV nsp14 residues N380, Y414, and N416 to explore the role of GpppA-interacting amino acids.

Unlike with rMHV_nsp14-D330A_, we were unable to recover a virus mutant containing an N416A substitution within the N7-MTase domain, suggesting that this residue, which was implicated in the formation of hydrogen bonds with the O atom at position C-3 of the guanosine ribose moiety, is critical for nsp14 function and virus replication. However, we were able to engineer and rescue a virus in which the Asn at position 380 was changed to Ala (rMHV_nsp14-N380A_). N386 of the SARS-CoV N7-MTase (corresponding to N380 in MHV) was shown to reside in immediate proximity to the atoms involved in methyl transfer by forming two hydrogen bonds with the guanine moiety of GpppA and was suggested to assist in orienting GpppA for catalysis (Fig. 1b) (32). We observed that initial rescue of this mutant from cloned DNA was prolonged, taking 5 days (compared to 1 to 2 days for wild-type rMHV) until sufficient recombinant virus had been obtained for further analysis (passage 1 virus) (see Fig. S1a in the supplemental material). By passage 2, replication of rMHV_nsp14-N380A_ was comparable to that of WT MHV (cytopathic effects occurred within 24 h), which prompted us to reassess the mutant virus genome sequence. As shown in Fig. S1a, the MHV nsp14-N380A mutant sequence rapidly changed to encode a serine residue at position 380, suggesting a strong selection toward regaining N7-MTase activity. Indeed, MHV nsp14-D380S, but not MHV nsp14-D380A, could complement mutants lacking N7-MTase activity in our previously established yeast genetic system. This system is based on Saccharomyces cerevisiae strain YBS40, which has a deletion in the *Abd1* locus (encoding *S. cerevisiae* cap MTase). Abd1 activity was restored after transformation with a plasmid encoding a functional N7-MTase. The capacity of viral proteins to substitute for yeast N7-MTase is indicated by selection of 5-fluoroorotic acid (5-FOA)-resistant colonies (18, 24) (Fig. S1b). Furthermore, using an established assay based on incubation of bacterially expressed recombinant MHV nsp14 and its mutants with substrate RNAs bearing an unmethylated GpppA-cap in the presence of the methyl donor SAM (18), we confirmed that MHV nsp14-380A was inactive, as expected, while the activity of MHV nsp14-380S was partially restored (Fig. S1c). Collectively, these results show that MHV nsp14 residues N416 and N380 are critical for virus replication and that the N380S mutant can partially recover wild-type MHV N7-MTase activity.

10.1128/mbio.03662-21.1 FIG S1 Revertant mutant of MHV_nsp14-N380_ and assessment of N7-MTase activity. (a) The figure shows the sequence of rMHV_nsp14-N380_ and compensatory mutations after passage. (b) Yeast complementation assay to examine the RNA cap guanosine N7-methylation function of wild-type (WT) nsp14 and MHV nsp14 mutants (with the N380A and N380S mutations and with D330A as a negative control). YBS40 was complemented by WT MHV nsp14 and the positive control, pYX232-HCM1. (c, d, e) GpppA-RNA was used to assay the methylation activities of nsp14 and its mutants as indicated. ***, *P < *0.001 (unpaired Student *t* test). Data are representative of three independent experiments (mean values ± SD). Download FIG S1, PDF file, 0.2 MB. Copyright © 2022 Pan et al. 2022 Pan et al. https://creativecommons.org/licenses/by/4.0/ This content is distributed under the terms of the Creative Commons Attribution 4.0 International license.

Next, we engineered a virus in which Y414 was mutated to Ala (Y414A) (34). Based on the structure of SARS-CoV nsp14, Y414 is predicted to be important but not necessary for optimal positioning of the purine moiety of the guanosine of GpppA for catalysis (Fig. S2a and b). The overall structure of MHV nsp14 complexed with substrate SAM and GpppA is predicted to be analogous to that of the SARS-CoV nsp14-substrate complex (Protein Data Bank [PDB] accession no. 5C8S) (Fig. S2a and S2b). Notably, mutation of nsp14-Y414H was previously shown to result in MHV attenuation in mice (35).

10.1128/mbio.03662-21.2 FIG S2 Structure of nsp14 and plaque size after rMHV or rMHV_nsp14-Y414A_ infection. (a) Overall structure of MHV nsp14 complexed with the substrate GpppA. The ExoN domain is shown in magenta, and the N7-MTase domain is shown in cyan. The substrate GpppA is shown as a stick figure. (b) Enlarged view of GpppA complexed with nsp14. The structure of MHV nsp14 was modeled by Swiss-Model, using SARS-CoV nsp14 complexed with the substrate SAM and with GpppA as the starting point. GpppA was put into the structure of MHV nsp14, and then the complex structure was optimized using Minimize in Phenix. Side chains of Y414 and other residues of MHV nsp14 stabilize the purine moiety of the guanosine of GpppA in an optimal position during catalysis. (c) Infection of murine L2 cells with rMHV or rMHV_nsp14-Y414A_ at an MOI of 1.0 (left) or 0.01 (right). The titers of virus were determined at the indicated time points. (d) Representative plaques formed by rMHV and rMHV_nsp14-Y414A_ at 24 hpi at 37°C. Data are representative of three independent experiments (mean values ± SD). Download FIG S2, PDF file, 0.6 MB. Copyright © 2022 Pan et al. 2022 Pan et al. https://creativecommons.org/licenses/by/4.0/ This content is distributed under the terms of the Creative Commons Attribution 4.0 International license.

We further investigated the role of Y414 in N7-MTase activity. The results showed that WT nsp14 and human N7-MTase (HCM1) but not nsp14-Y414A, nsp14-Y414H, or nsp14-D330A (corresponding to SARS-CoV D331 and previously shown to lack N7-MTase activity) (24) complemented the growth of YBS40 (Fig. 1c), suggesting that MHV nsp14-Y414A, nsp14-Y414H, and nsp14-D330A lacked N7-MTase activity. As recently described (34) and also shown in Fig. S1d, these results were confirmed using bacterially expressed recombinant MHV nsp14 and its mutants, which were incubated *in vitro* with substrate RNAs bearing an unmethylated GpppA-cap in the presence of the methyl donor SAM by thin-layer chromatography (TLC) (Fig. 1d) and by measuring ^3^H incorporation (Fig. S1d). WT nsp14 but not nsp14-Y414A, nsp14-Y414H, or nsp14-D330A was able to methylate the incompletely methylated substrate, indicating that Y414, in addition to D330 of MHV nsp14, was necessary for N7-MTase activity. Subsequently, we investigated the N7-MTase activities of these identical mutations in the context of nsp14 of SARS-CoV-2. The results showed that SARS-CoV-2 nsp14-Y420H, nsp14-Y420A, and nsp14-D331A could not complement the growth of YBS40 as exogenous N7-MTase (Fig. 1c, bottom) and that WT nsp14 but not nsp14-Y420H, nsp14-Y420A, or nsp14-D331A protein was able to methylate the incompletely methylated substrate (Fig. 1f). These results further confirmed that this highly conserved tyrosine at position 414/420 in nsp14 was necessary for its N7-MTase activity in MHV/SARS-CoV-2.

Next, we investigated the effect of CoV nsp14 N7-MTase activity on virus replication, using rMHV_nsp14-Y414A_. Even though nsp14-Y414A lacks N7-MTase activity when measured *in vitro*, we detected similar replication kinetics, plaque sizes, and morphologies when rMHV_nsp14-Y414A_- and WT-MHV-infected 17CL-1 or L2 cells were compared (Fig. 1e and Fig. S2d). Similar results were obtained when virus replication kinetics were measured in L2 cells (Fig. S2c) and as described previously (34). After five serial passages in culture cells, the nsp14 gene of the rMHV_nsp14-Y414A_ genome was sequenced. No additional mutations were observed. To evaluate the methylation status of viral RNA, we enriched total poly(A)-containing RNAs from cells infected with rMHV or rMHV_nsp14-Y414A_ and subjected the RNA to an *in vitro* methylation assay using [^3^H]SAM and nsp14 (N7-MTase) or the nsp10/nsp16 complex (2′-O-MTase) (10). The results showed that, when exposed to WT nsp14 but not WT nsp10/nsp16, cellular mRNA from rMHV_nsp14-Y414A_-infected cells exhibited increased ^3^H methylation compared to that of cellular mRNA obtained from rMHV-infected cells (Fig. 1g). Thus, as expected, viral mRNA obtained from rMHV_nsp14-Y414A_-infected cells could be methylated by WT nsp14, consistent with absent or incomplete cap-0 N7-methylation (GpppN-RNA). Taken together, the results demonstrated that the nsp14-Y414A mutation did not decrease replication in cultured cells even though levels of cap-0 N7-methylation were below the level of detection.

### Infection with rMHV_nsp14-Y414A_ upregulates IFN-β expression and results in reduced viral replication in primary immune cells.

**FIG 2:**
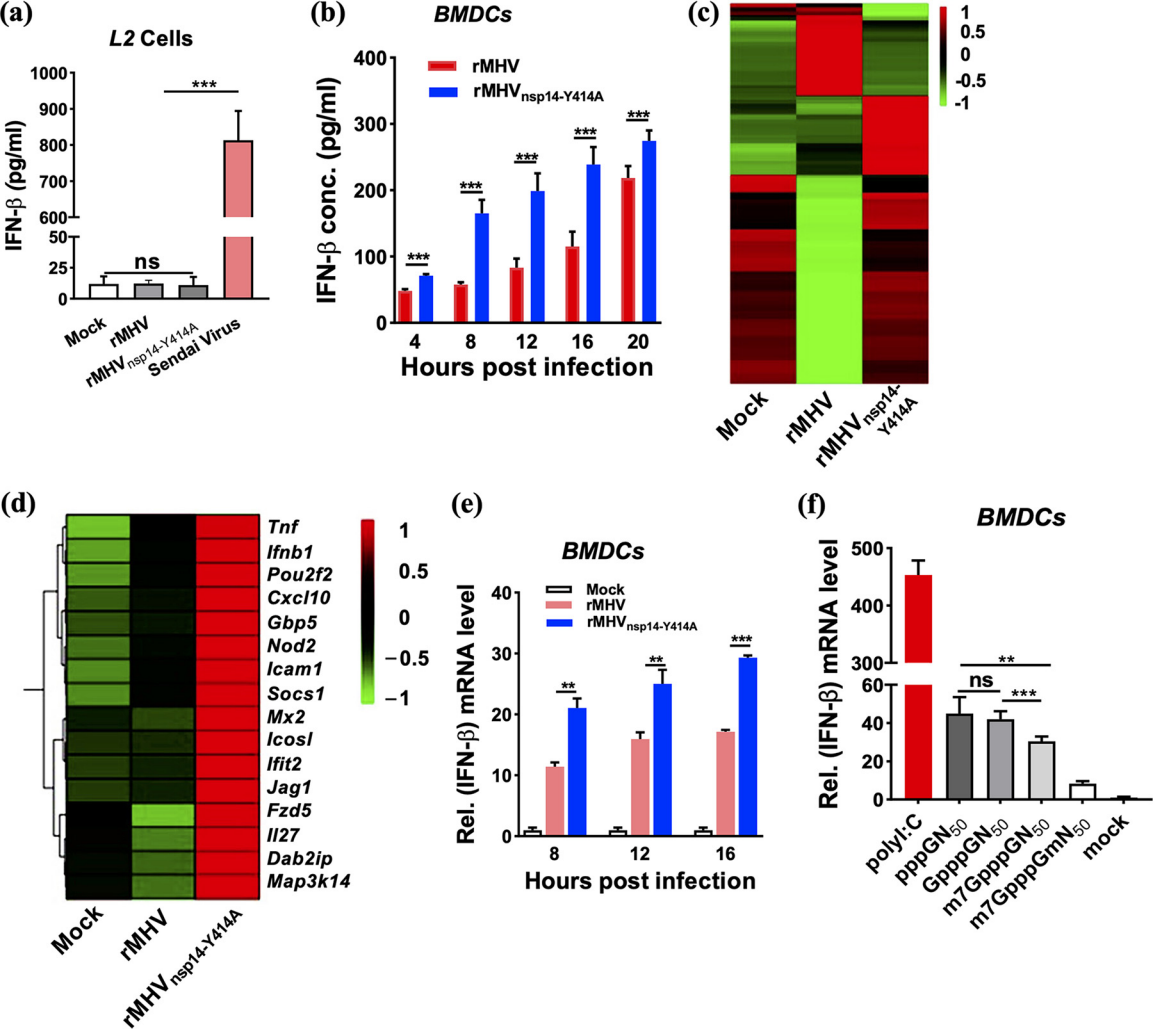
rMHV_nsp14-Y414A_-induced IFN-β expression and IFN signaling in primary cells. (a) Levels of IFN-β in L2 cell supernatants obtained at 12 hpi. (b) Levels of IFN-β in supernatants of infected bone marrow-derived dendritic cells (BMDCs) at the indicated time points. (c, d) RNA isolated from BMDCs infected with rMHV or rMHV_nsp14-Y414A_ at 8 hpi and subjected to microarray analysis. (c) The heatmap shows 257 genes differentially expressed (DE) between rMHV_nsp14-Y414A_-infected BMDCs and mock- or rMHV-infected BMDCs. (d) Differentially expressed immune response genes. (e) Two micrograms of viral RNA were isolated from purified virions harvested from the supernatant and was transfected into BMDCs. Cells were analyzed for *Ifn-β* mRNA at 8, 12, and 16 hpi. (f) Fifty nanomolar RNA fragments (51 nt) from the 5′-UTR of the MHV-A59 genome with the indicated 5′-end sequences were transfected into BMDCs and analyzed for *Ifn-β* mRNA by qRT-PCR at 20 h posttransfection. Poly(I·C) was a positive control. ns, not significant; **, *P < *0.01; ***, *P < *0.001 (unpaired Student *t* test). Data are representative of three independent experiments (mean values ± SD). Rel., relative.

MHV infection induces IFN-I production in primary dendritic cells and macrophages but not in cell lines (36–39). To further assess the effect of the rMHV mutant harboring nsp14-Y414A on IFN-I induction, we infected L2 and bone marrow-derived dendritic cells (BMDCs) with rMHV or rMHV_nsp14-Y414A_. We detected no IFN-β in the supernatant of L2 cells infected with rMHV or rMHV_nsp14-Y414A_ (Fig. 2a and Fig. S3). However, both rMHV and rMHV_nsp14-Y414A_ induced IFN-β expression in BMDCs. Moreover, rMHV_nsp14-Y414A_ induced significantly higher IFN-β levels than rMHV (Fig. 2b).

10.1128/mbio.03662-21.3 FIG S3 rMHV and rMHV_nsp14-Y414A_ induce IFN-I equivalently in L2 cells. L2 cells were infected with rMHV, rMHV_nsp14-Y414A_, or Sendai virus as a positive control. Cell culture supernatants were collected and clarified by centrifugation. After UV radiation, inactivated culture supernatants were applied to L2 cells for 2 h. L2 cells were infected with VSV-GFP at an MOI of 1.0. After 24 h of cell culture, virus replication was evaluated by monitoring GFP expression by fluorescence microscopy, as a measure of IFN-β secretion. Data are representative of 3 independent experiments. Of note, no fluorescent signal was detected in any Sendai virus-infected sample. Download FIG S3, PDF file, 0.2 MB. Copyright © 2022 Pan et al. 2022 Pan et al. https://creativecommons.org/licenses/by/4.0/ This content is distributed under the terms of the Creative Commons Attribution 4.0 International license.

To determine whether rMHV_nsp14-Y414A_ broadly induced an augmented innate immune response, we analyzed host transcriptomes in BMDCs infected with rMHV or rMHV_nsp14-Y414A_ using high-throughput transcriptome sequencing (RNA-seq) (Fig. 2c). Expression levels of innate immune-related genes at 6 h postinfection (hpi) were significantly upregulated in rMHV_nsp14-Y414A_ compared to levels in rMHV-infected BMDCs (Fig. 2d). To exclude a direct effect of the mutation on IFN induction, we engineered lentiviruses expressing Y414A and WT nsp14 (Fig. S4a) and used them to transduce BMDCs. As shown in Fig. S4b, neither WT nor mutant nsp14 expressed in isolation induced IFN-β production, and neither protein could inhibit IFN-β mRNA expression in Sendai virus (SeV)-infected BMDCs.

10.1128/mbio.03662-21.4 FIG S4 The nsp14 and nsp14-Y414A proteins do not affect the expression of IFN-β. (a) Viral proteins nsp14 and nsp14-Y414A were introduced into lentivirus vectors, and expression was confirmed by Western blot analysis with anti-His antibodies. (b) BMDCs were infected with lentivirus expressing nsp14 or nsp14-Y414A and were unstimulated (uns) or stimulated with SeV for 8 h. The mRNA of *Ifn-β* was detected by qRT-PCR. ns, not significant (unpaired Student *t* test). Data are representative of three independent experiments (mean values ± SD). Download FIG S4, PDF file, 0.08 MB. Copyright © 2022 Pan et al. 2022 Pan et al. https://creativecommons.org/licenses/by/4.0/ This content is distributed under the terms of the Creative Commons Attribution 4.0 International license.

To assess whether N7-unmethylated RNA was incorporated into virions, we prepared viral RNA from purified virions harvested from the supernatants of rMHV and rMHV_nsp14-Y414A_-infected neuron-2A cells. Two micrograms of RNA was transfected into BMDCs and analyzed for IFN-β mRNA at 8, 12, and 16 h posttransfection. As shown in Fig. 2e, the RNA of rMHV_nsp4-Y414A_ triggered a significantly increased accumulation of IFN-β mRNA compared to that of rMHV, indicating that N7-unmethylated viral RNA (GpppN-RNA) stimulated the expression of IFN-β at early times after transfection. Moreover, we transfected synthetic 51-nucleotide (nt) MHV 5′ untranscribed region (5′-UTR) RNA with different 5′-end structures (pppGN_50_, GpppGN_50_, m7GpppGN_50_, and m7GpppGmN_50_) into BMDCs and analyzed IFN-β mRNA expression at 20 h posttransfection (Fig. 2f). All of the RNAs induced the transcription of IFN-β mRNA to various degrees. Synthetic RNAs pppGN_50_ and GpppGN_50_ induced equivalent amounts of IFN-β mRNA, which were significantly higher than levels induced by m7GpppGN_50_. Among all of the 5′-UTR RNAs, m7GpppGmN_50_ induced the least IFN-β mRNA. Collectively, these results indicated that N7-methylation plays an important role in IFN-I induction, independently of 2′-O-methylation.

**FIG 3:**
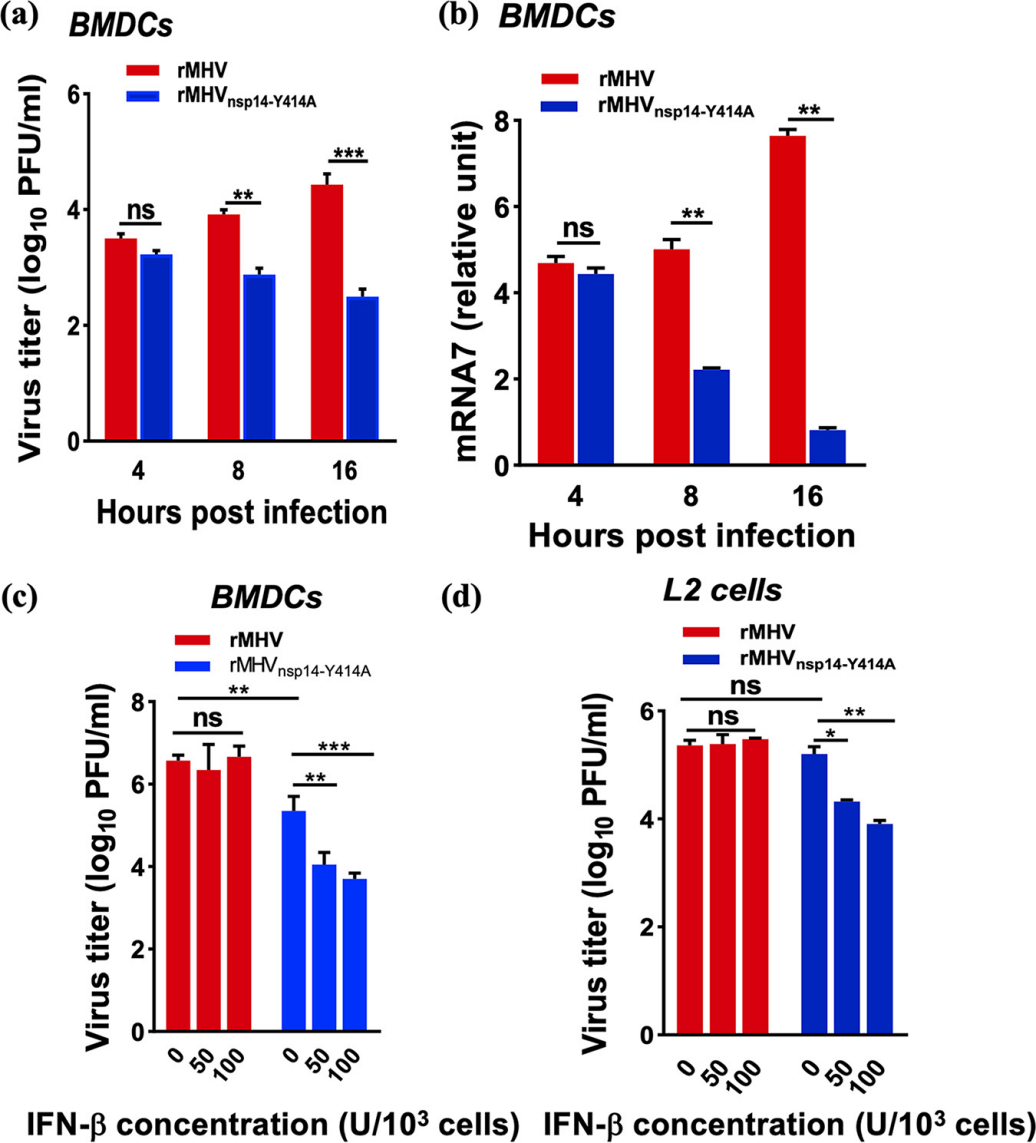
Diminished replication of rMHV_nsp14-Y414A_ in BMDCs and increased IFN-β sensitivity. (a, b) Infection of BMDCs with rMHV or rMHV_nsp14-Y414A_ at an MOI of 0.1. (a, b) Virus titers at the indicated time points were assessed by plaque assay (a) and by qRT-PCR with primers useful for detecting MHV mRNA7 (nucleocapsid gene) (b). Levels of mRNA7 relative to that of β-actin mRNA are expressed as 2^–ΔCT^[ΔCT = CT_(mRNA7)_ − CT_(β-actin)_], where CT is the threshold cycle. (c, d) BMDCs (c) and L2 cells (d) were treated with the indicated dosages of IFN-β for 4 h before and after infection with rMHV or rMHV_nsp14-Y414A_ (MOI of 1). Virus titers were measured 8 h after infection. ns, not significant; *, *P < *0.05; **, *P < *0.01; ***, *P < *0.001 (unpaired Student *t* test). Data are representative of three independent experiments (mean values ± SD).

Next, we measured virus titers and viral mRNA concentration in BMDCs infected with rMHV and rMHV_nsp14-Y414A_. Virus titers (Fig. 3a) and viral mRNA levels (Fig. 3b) in rMHV_nsp14-Y414A_-infected cells were significantly decreased compared to those in BMDCs infected with WT rMHV. Of note, N7-unmethylated virion RNA likely induces a rapid innate immune response upon initial infection of uninfected cells, contributing to an attenuated virus phenotype.

Previous studies identified that the absence of 2′-O-methylation resulted in both IFN-I induction and increased sensitivity to IFN-induced proteins with tetratricopeptide repeats (IFIT) (5). To assess whether the decrease in N7-methylation also rendered virus more sensitive to IFN-I, BMDCs and L2 cells were pretreated with different doses of recombinant murine IFN-β for 4 h, and virus titers were measured at 8 hpi. Virus titers were significantly decreased in rMHV_nsp14-Y414A_-infected BMDCs compared to levels in rMHV-infected BMDCs (Fig. 3c) and L2 cells (Fig. 3d) in a dose-dependent manner. Further investigation will be required to determine whether this sensitivity is mediated by IFIT or other IFN-I-induced proteins.

### MDA5 and RIG-I recognize RNA harvested from rMHV_nsp14-Y414A_-infected cells.

**FIG 4:**
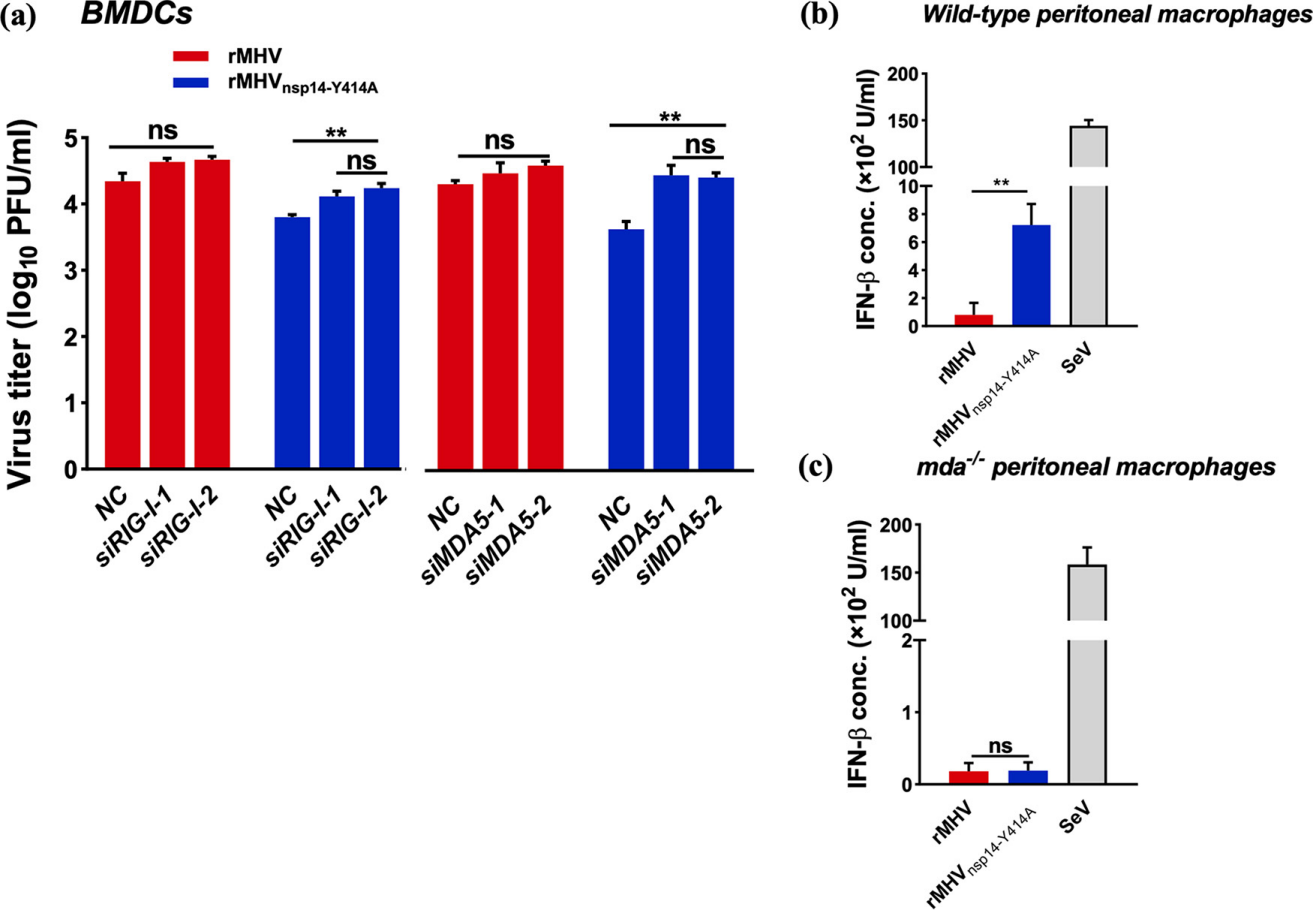
IFN-β expression in rMHV_nsp14-Y414A_-infected primary cells is dependent upon RLR activation. (a) BMDCs transfected with siRNA targeting RIG-I (left) or MDA5 (right) for 24 h, prior to infection with rMHV or rMHV_nsp14-Y414A_ at an MOI of 0.1. Virus titers were measured by plaque assay at 16 hpi. (b, c) Infection of C57BL/6 (b) or *MDA5*-deficient (*mda5*^−/−^) (c) peritoneal macrophages with rMHV, rMHV_nsp14-Y414A_, or Sendai virus (SeV) as a positive control at an MOI of 1. The level of IFN-β in the cell supernatant was measured by ELISA at 12 hpi. (d, e) Infection of peritoneal macrophages derived from C57BL/6 (d) or *mda5*^−/−^ (e) mice with rMHV or rMHV_nsp14-Y414A_ at an MOI of 0.0001. Virus titers were measured by plaque assay. ns, not significant; *, *P < *0.05; **, *P < *0.01; ***, *P < *0.001 (unpaired Student *t* test). Data are representative of three independent experiments (mean values ± SD).

For RNA viruses, the main PRRs in the cytosol are RIG-I-like receptors (RLRs), which include RIG-I and MDA5 (40). To determine whether one or both of these molecules are involved in the recognition of N7-unmethylated RNA, we treated BMDCs with lentiviruses expressing RIG-I- or MDA5-specific short hairpin RNAs (shRNAs), respectively (Fig. S5). We found that after knockdown of RIG-I or MDA5, significantly increased titers of rMHV_nsp14-Y414A_ were observed (*P < *0.01), while rMHV titers were unchanged (*P > *0.05) from titers in negative controls (Fig. 4a). We also investigated IFN-β production in WT and *mda5*^−/−^ peritoneal macrophages infected with rMHV or rMHV_nsp14-Y414A_. In WT peritoneal macrophages, rMHV_nsp14-Y414A_ induced significantly increased IFN-β expression compared to that induced by rMHV (Fig. 4b). However, in *mda5*^−/−^ peritoneal macrophages, no IFN-β was induced by rMHV or rMHV_nsp14-Y414A_ (Fig. 4c). Collectively, these results demonstrated that MDA5 and RIG-I are involved in the recognition of N7-unmethylated RNA and thereby restricted the replication of rMH_Vnsp14-Y414A_. However, based on our analysis of *mda5*^−/−^ macrophages and a previous publication (41), MDA5 is likely the more important sensor in MHV-infected myeloid cells.

10.1128/mbio.03662-21.5 FIG S5 Knockdown of RIG-I and MDA5 in BMDCs. BMDCs were transfected with negative-control siRNA (si-NC), *RIG-I*-targeting siRNAs (siRIG-I-1 and siRIG-I-2), or *mda5*-targeting siRNAs (siMDA5-1 and siMDA5-2). The cells were collected 24 h posttransfection and subjected to qRT-PCR to evaluate the efficiency of siRNAs. *, *P < *0.05; **, *P < *0.01 (unpaired Student *t* test). Data are representative of three independent experiments (mean values ± SD). Download FIG S5, PDF file, 0.04 MB. Copyright © 2022 Pan et al. 2022 Pan et al. https://creativecommons.org/licenses/by/4.0/ This content is distributed under the terms of the Creative Commons Attribution 4.0 International license.

### rMHV_nsp14-Y414A_ exhibits reduced pathogenicity in mice.

**FIG 5:**
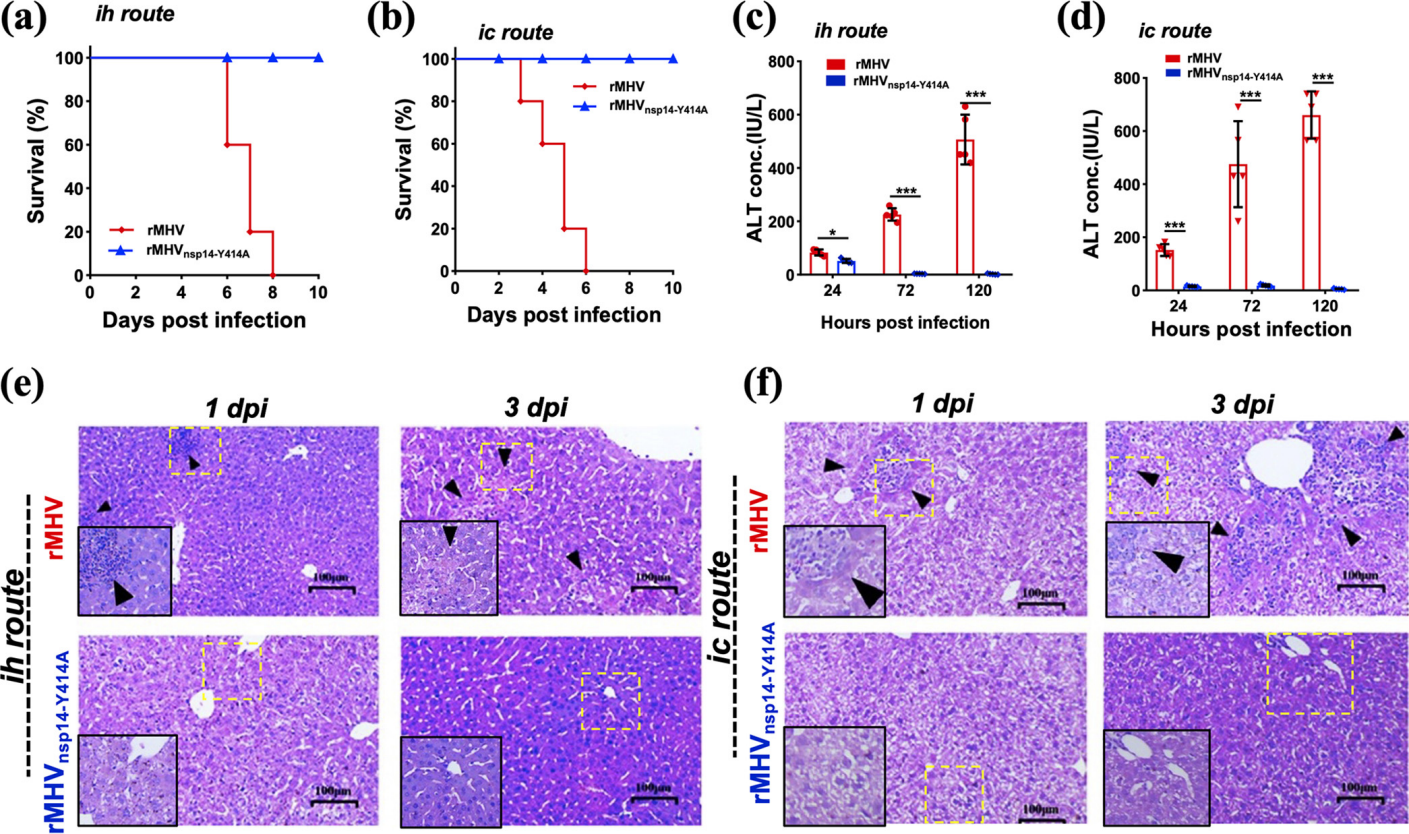
rMHV_nsp14-Y414A_ exhibits reduced pathogenicity in mice. Four-week-old C57/BL6 mice (*n *= 5) were inoculated intrahepatically (i.h.) (a, c, e) or intracranially (i.c.) (b, d, f) with 10^4^ PFU of rMHV or rMHV_nsp14-Y414A_. (a, b) Mortality was monitored daily. (c, d) Levels of serum ALT were measured at 24, 72, and 120 hpi. (e, f) Livers of rMHV- or rMHV_nsp14-Y414A_- infected mice were stained with hematoxylin-eosin and examined for pathological changes at 1 and 3 days p.i. Arrowheads indicate inflammatory foci and tissue damage in livers of the infected mice. The inset shows higher magnification. Data are representative of two independent experiments (mean values ± SD). IU, international units.

We previously showed that rMHV_nsp14-Y414A_ was attenuated in mice after intrahepatic (i.h.) inoculation (34). To confirm that nsp14 N7-MTase activity was required for maximal virulence in mice, we infected 4-week-old C57BL/6 mice by intracranial (i.c.) inoculation with rMHV or rMHV_nsp14-Y414A_ and compared the results to mice infected via i.h. inoculation. All mice infected with rMHV by either route died by day 8 postinoculation (p.i.), while all mice infected with rMHV_nsp14-Y414A_ survived (Fig. 5a and b). Measurement of virus titers showed that rMHV_nsp14-Y414A_ was completely cleared from the liver and brain by 5 days p.i. (dpi) (Table 1). Of note, rMHV readily spreads to and infects the liver after i.c. inoculation, but this did not occur in mice infected with rMHV_nsp14-Y414A_ (Table 1). Moreover, levels of alanine transaminase (ALT) in sera of rMHV_nsp14-Y414A_-infected mice were transiently elevated at 24 h p.i. (hpi) through both routes of inoculation (Fig. 5c and d). In contrast, serum ALT levels were elevated for the entire course of the infection in mice infected with rMHV by either i.h. or i.c. inoculation (Fig. 5c and d). Histopathological analysis displayed necrotic foci and parenchymal inflammation in the livers of mice infected with rMHV at day 3 p.i. In contrast, no obvious histopathological changes were observed in the livers of rMHV_nsp14-Y414A_-infected mice (Fig. 5e and f). These results demonstrate that rMHV_nsp14-Y414_ displayed a highly attenuated phenotype in mice infected by the i.c. route, similar to what was observed after i.h. inoculation, and that rMHV_nsp14-Y414A_ lost its ability to spread from brain to liver after i.c. inoculation.

**TABLE 1:** Virus titers in rMHV- or rMHV_nsp14-Y414A_-infected brains or livers of 4-week-old *Ifnar*^+/+^ C57/BL6 mice by intracranial or intrahepatic inoculation^*a*^

		Titer by intrahepatic route		Titer by intracranial route	
Virus strain	hpi	Liver	Brain	Liver	Brain
rMHV	24	3.46 ± 0.15	ND	3.46 ± 0.43	3.74 ± 0.16
	48	4.17 ± 0.28	ND	4.26 ± 0.43	3.80 ± 0.65
	72	4.18 ± 0.34	ND	5.06 ± 0.50	5.00 ± 0.29
	120	3.74 ± 0.41	ND	D	D

rMHV_nsp14-Y414A_	24	1.33 ± 0.32	ND	ND	2.42 ± 0.33
	48	ND	ND	ND	3.30 ± 0.21
	72	ND	ND	ND	1.06 ± 0.49
	120	ND	ND	ND	ND

a Virus titers are expressed as log_10_ numbers of PFU per milliliter. ND, not detected; D, death.

### Early IFN-I expression and transcriptomic characteristics of rMHV- and rMHV_nsp14Y414A_-infected mice.

**FIG 6:**
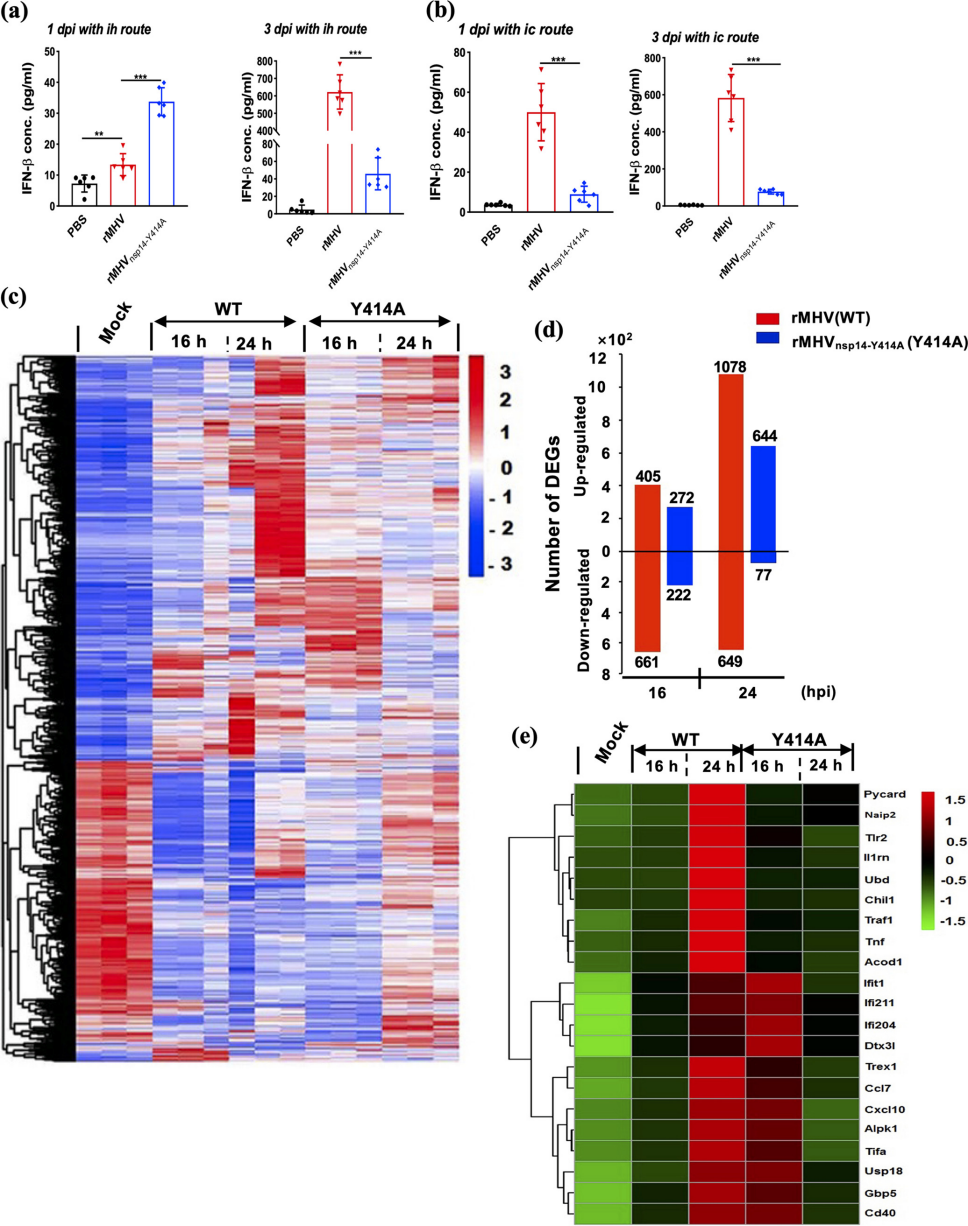
Early expression of IFN-β in sera and transcriptomic analysis in livers of rMHV- and rMHV_nsp14Y414A_-infected C57BL/6 mice. For IFN-β expression, IFN-β in the sera of 4-week-old C57BL/6 mice (six mice per each group) infected with rMHV or rMHV_nsp14-Y414A_ was assayed at the indicated time points by ELISA after i.h. (a) or i.c. (b) inoculation. Data are representative of two independent experiments (mean values ± SD). For transcriptome analysis, 4-week-old C57BL/6 mice (3 mice per each group) were intrahepatically inoculated with rMHV and rMHV_nsp14Y414A_ (7.5 × 10^5^ PFU of virus per mouse). After 16 h and 24 h of infection, liver tissues of mice were collected for transcriptome sequencing. (c) A heatmap depicting the expression pattern of genes significantly upregulated and downregulated in each group is shown. (d) Numbers of upregulated and downregulated genes differentially expressed between infected mice and naive mice. Criteria used for differential expression analysis are a *q* value of <0.05 and a |log_2_ fold change| of >1. (e) Differentially expressed immune response-related genes.

As our previous results showed that rMHV_nsp14-Y414A_ induced higher IFN-I production in BMDCs and was more sensitive to IFN treatment *in vitro* (Fig. 2b and 3c), we next measured IFN levels in the sera of infected mice. As shown in Fig. 6a, IFN-β levels were higher in the sera of mice infected i.h. with rMHV_nsp14-Y414A_ at day 1 p.i. than in mice infected with rMHV, consistent with the *in vitro* results. However, by day 3 p.i., levels were higher in rMHV-infected mice, likely reflecting the elevated virus titers detected in these mice (Table 1). In contrast, IFN-β titers were higher in rMHV than in rMHV_nsp14-Y414A_-infected mice after i.c. inoculation at both days 1 and 3 p.i. (Fig. 6b), perhaps reflecting the lack of hepatic involvement after rMHV_nsp14-Y414A_ infection.

To further understand the mechanism of enhanced innate immune response induced by rMHV_nsp14-Y414A_, the transcriptome profiles in livers of the experimental mice were analyzed by RNA-seq (Fig. 6c to e). We first identified genes differentially expressed (DE) between rMHV- and rMHV_nsp14-Y414A_-infected mice and naive mice for each time point using the following criteria: a >2-fold change and a false-discovery rate [*q*] of <0.05, as determined by LIMMA’s empirical Bayes moderated *t* test. In total, 2,554 genes were differentially expressed in at least one experiment group, with 1,520 upregulated and 1,034 downregulated genes (Fig. 6c and Table S1). In mice infected with rMHV, we detected 1,067 and 1,078 DE genes at 16 and 24 hpi, respectively. For rMHV_nsp14-Y414A_-infected mice, 494 and 721 genes were differentially regulated at 16 and 24 hpi (Fig. 6c and Table S1). Gene ontology (GO) annotation showed that in mice infected with rMHV, DE genes were involved mainly in metabolic processes, immune responses, and biosynthetic processes, while DE genes in mice infected with rMHV_nsp14-Y414A_ mediated mainly regulation of the immune response, IFN type I production, and responses and PRR signaling pathways (Table S2). We next focused on genes related to host immune responses, including those noted to exhibit differential expression in infected BMDCs (Fig. 2d). We found that several genes, including *Tlr2*, *TNF*, and *Traf1*, were preferentially upregulated at 16 h p.i. in rMHV_nsp14-Y414A_- compared to rMHV-infected mice, but by 24 h, these genes exhibited substantial upregulation in rMHV-infected mice (Fig. 6e). Delayed expression of these proinflammatory molecules may contribute to greater disease in MHV- than in rMHV_nsp14-Y414A_-infected mice. However, identification of proteins most important for enhanced disease will require further investigation.

10.1128/mbio.03662-21.6 TABLE S1 Total DE genes and DE genes from four experimental groups. Download Table S1, XLSX file, 0.4 MB. Copyright © 2022 Pan et al. 2022 Pan et al. https://creativecommons.org/licenses/by/4.0/ This content is distributed under the terms of the Creative Commons Attribution 4.0 International license.

10.1128/mbio.03662-21.7 TABLE S2 Top 20 GO annotation of the DE genes from the total and from four experimental groups. Download Table S2, XLSX file, 0.03 MB. Copyright © 2022 Pan et al. 2022 Pan et al. https://creativecommons.org/licenses/by/4.0/ This content is distributed under the terms of the Creative Commons Attribution 4.0 International license.

### Restriction of rMHV_nsp14-Y414A_ replication is restored in *Ifnar^−/−^* BMDCs and mice.

**FIG 7:**
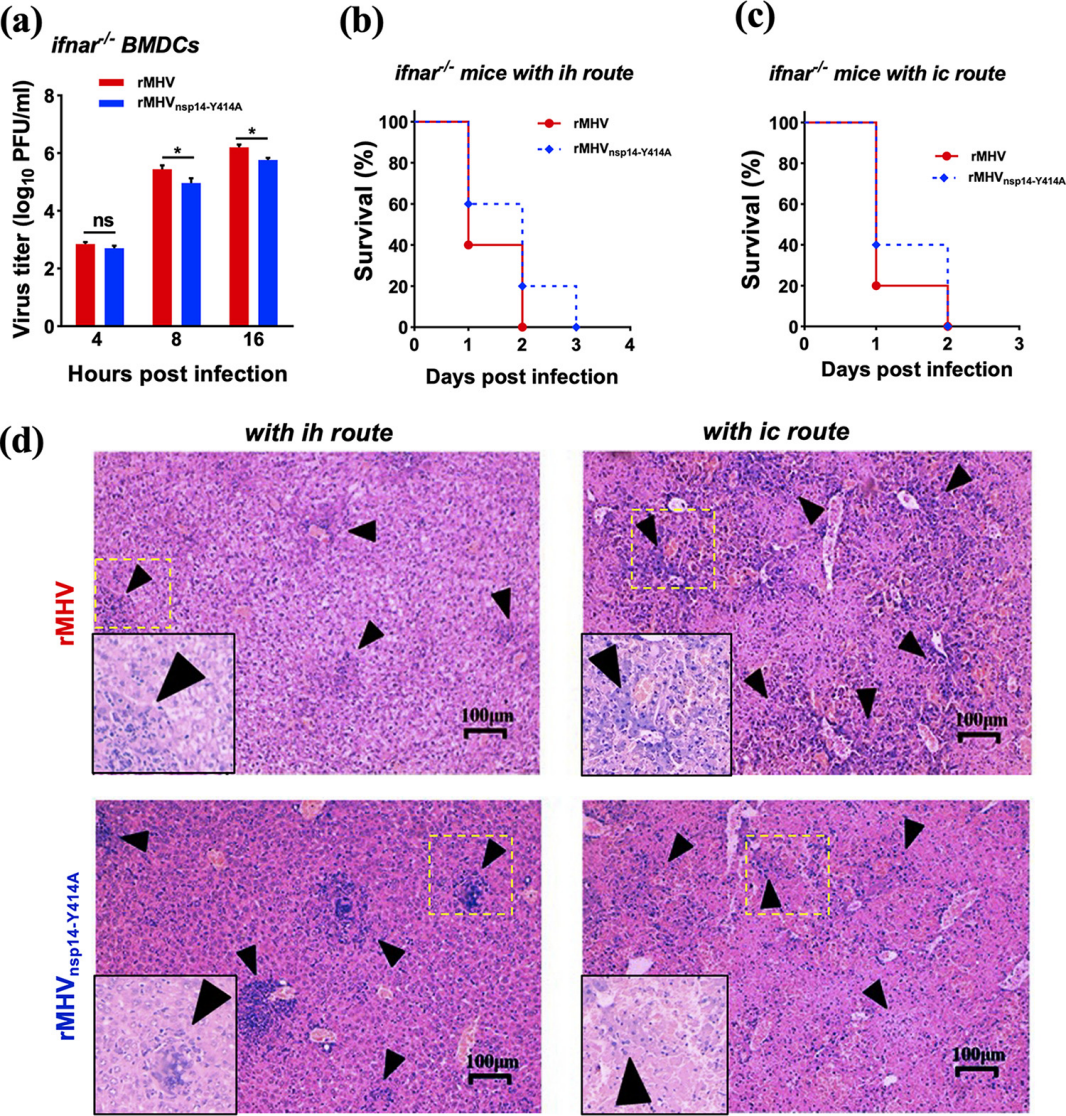
Restriction of rMHV_nsp14-Y414A_ replication is restored in *Ifnar*^−/−^ BMDCs and mice. (a) Infection of *Ifnar*^−/−^ BMDCs with rMHV or rMHV_nsp14-Y414A_ at an MOI of 0.1. Virus titers at the indicated time points were assessed by plaque assay. (b, c) Four-week-old *Ifnar*^−/−^ mice were infected i.h. (b) or i.c. (c) and monitored for survival. (d) Histopathological analysis of liver tissues of *Ifnar*^−/−^ mice infected with rMHV or rMHV_nsp14-Y414A_ at 1 dpi. Arrowheads indicate large perivascular infiltrates detected at 1 dpi in rMHV- or rMHV_nsp14-Y414A_-infected mice. (b to d) Data are representative of 10 mice per group. ns, not significant; *, *P < *0.05 (unpaired Student *t* test). Data are representative of two independent experiments (mean values ± SD).

Our results suggest that IFN has a key role in suppressing rMHV_nsp14-Y414A_ replication in infected mice. To assess the role of IFN-I in restricting rMHV_nsp14-Y414A_ replication directly, we infected BMDCs lacking IFN-I receptor (*Ifnar*^−/−^) expression with rMHV or rMHV_nsp14-Y414A_ and measured virus titers. As shown in Fig. 7a, virus titers were modestly higher in rMHV- than in rMHV_nsp14-Y414A_-infected cells, but the difference in titers was less than observed in WT BMDCs (Fig. 3a). To confirm the role of IFN-I in diminished rMHV_nsp14-Y414A_ pathogenicity, *Ifnar*^−/−^ mice were infected i.h. or i.c. with rMHV or rMHV_nsp14-Y414A_. Mice infected with both viruses after inoculation by either route died (Fig. 7b and c), in marked contrast to results obtained after infection of C57BL/6 mice (Fig. 5a and b). Additionally, virus titers were similar in the brains and livers of *Ifnar*^−/−^ mice infected with rMHV or rMHV_nsp14-Y414A_. Of note, rMHV_nsp14-Y414A_ spread to the brain after i.h. infection of *Ifnar*^−/−^ mice, while it remained confined to the liver in C57BL/6 mice (Tables 1 and 2), likely contributing to death. Histopathological analysis revealed necrotic foci and inflammatory cell infiltration in the liver parenchyma in rMHV- and rMHV_nsp14-Y414A_-infected *Ifnar*^−/−^ mice, although histological changes were more extensive in rMHV-infected mice (Fig. 7d). Together, these results indicate that rMHV_nsp14-Y414A_ attenuation is associated with enhanced IFN signaling.

**TABLE 2:** Virus titers in rMHV- or rMHV_nsp14-Y414A_-infected brains or livers of 4-week-old *Ifnar^−/−^* C57/BL6 mice by intracranial or intrahepatic inoculation^*a*^

		Titer by intrahepatic route		Titer by intracranial route	
Virus strain	hpi	Liver	Brain	Liver	Brain
rMHV	24	6.08 ± 0.08	2.53 ± 0.23	6.25 ± 0.05	5.47 ± 0.12
	48	D	D	D	D
	72				

rMHV_nsp14-Y414A_	24	4.31 ± 0.39	1 ± 0.11	5.03 ± 0.33	5.61 ± 0.12
	48	D	D	D	D
	72				

a Virus titers are expressed as log_10_ numbers of PFU per milliliter. D, death.

### rSARS-CoV-2_nsp14-Y420A_ exhibits the attenuated phenotype and protects mice against lethal SARS-CoV-2 challenge.

**FIG 8:**
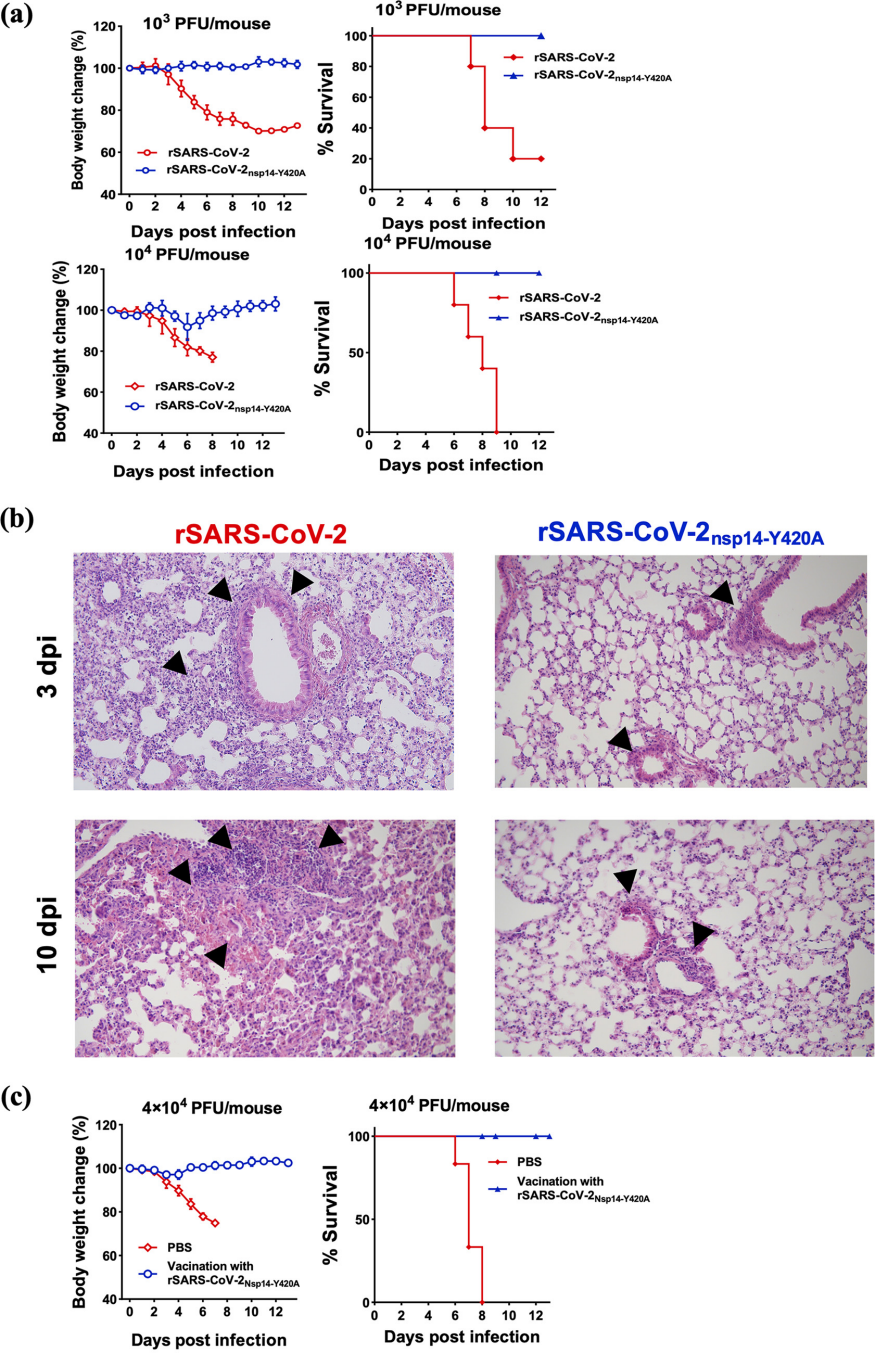
rSARS-CoV-2_nsp14-Y414A_ is attenuated and protects mice against lethal SARS-CoV-2 challenge. (a) Eight-week-old K18-hACE2 transgenic mice were intranasally (i.n.) infected with 10^3^ PFU (upper) or 10^4^ PFU (bottom) of rSARS-CoV-2 or rSARS-CoV-2_nsp14-Y420A_, respectively (*n *= 5). Body weights (left) and mortality (right) were monitored daily. (b) Lungs of mice infected with rSARS-CoV-2 or rSARS-CoV-2_nsp14-Y420A_ were stained with hematoxylin-eosin and examined for pathological changes at 3 and 10 days p.i. Black arrows indicated inflammatory cell infiltrates. (c) Eight-week-old K18-hACE2 transgenic mice were inoculated with 1 × 10^4^ PFU of rSARS-CoV-2_nsp-Y420A_ or phosphate-buffered saline (PBS) (*n *= 5). At 40 dpi, animals were i.n. challenged with a lethal dose of 5 × 10^4^ PFU of rSARS-CoV-2. Weight change (left) and survival (right) were observed daily. Means with SD are shown (*n *= 5).

Our results and those of others demonstrated increased IFN production after infection with virus defective in N7-MTase function (33, 42). These results suggested that these attenuated viruses might be useful vaccine candidates, especially in preventing/decreasing SARS-CoV-2, the cause of the ongoing COVID-19 pandemic. Therefore, using the recombinant SARS-CoV-2 engineered as described in Materials and Methods, we next infected mice with rSARS-CoV-2 and rSARS-CoV-2_nsp14-Y420A_ and evaluated the virulence and pathogenicity of rSARS-CoV-2_nsp14-Y420A_ in 8-week-old K18-human ACE2 (hACE2) mice, which are highly susceptible to SARS-CoV-2 infection (43). Mice inoculated with 10^3^ PFU of rSARS-CoV-2 exhibited substantial weight loss, and 80% of animals died by 12 dpi (Fig. 8a, top). In contrast, rSARS-CoV-2_nsp14-Y420A_-infected mice showed modest weight loss, and all mice survived the infection. Even when inoculated with a higher dose (10^4^ PFU), rSARS-CoV-2_nsp14-Y420A_-infected mice showed only minor weight loss (about 10%), and all recovered. Unsurprisingly, all mice succumbed to 10^4^ PFU of rSARS-CoV-2 infection by 9 dpi (Fig. 8a, bottom). Histopathological analysis showed that pathological changes in lung tissues of mice infected with rSARS-CoV-2 were present at day 3 p.i. (Fig. 8b). These changes included thickening of interalveolar septa and inflammatory infiltration into peribronchial and perivascular areas. Only minimal pathological changes were noted in mice infected with rSARS-CoV-2_nsp14-Y420A_. By day 10 p.i., most mice infected with rSARS-CoV-2 died, but extensive hemorrhage and cellular infiltration were detected in the mice that survived. In marked contrast, only a modest amount of peribronchial cell infiltration was detected in rSARS-CoV-2_nsp14-Y420A_-infected mice. These results indicated that the Y420A mutation, which resulted in an inactivated nsp14 N7-MTase, also significantly decreased the virulence and pathogenicity of SARS-CoV-2.

To explore the attenuated mutant as a potential vaccine candidate, we evaluated the protection against SARS-CoV-2 infection induced by rSARS-CoV-2_nsp14-Y420A_ vaccination in mice. A single dose of rSARS-CoV-2_nsp14-Y420A_ delivered intranasally conferred complete protection against lethal SARS-CoV-2 challenge (Fig. 8c), indicating that rSARS-CoV-2_nsp14-Y420A_ may be a novel vaccine candidate.

## DISCUSSION

Here, we show that N7-methylation of viral RNA is important for CoV immune evasion and viral pathogenicity. First, we used purified enzymes, a yeast complementation assay, and a murine model of coronavirus infection to extend our previous report (34) that demonstrated that MHV-nsp14 acted as an N7-MTase. We also showed that similar results were obtained when wild-type and mutant SARS-CoV-2 nsp14s were analyzed, indicating that the critical residues of MHV and SARS-CoV-2 are highly conserved (Fig. 1). Our study was instructed by, and built upon, previous work that focused on structural and biochemical aspects of coronaviral nsp14-encoded N7-MTases (18, 31, 32). We specifically targeted residues of the MHV-nsp14 N7-MTase and SARS-CoV-2 N7-MTase domains that were proposed to have critical roles in substrate binding and methyl transfer. Our results confirm a previously noted discrepancy between apparent requirements of coronaviral N7-MTase activity *in vitro* and in the context of virus infection (33). Mutant MHV proteins nsp14-Y414A, nsp14-N416A, and nsp14-N380A were all defective in biochemical assays assessing N7-methyltransferase activity and in yeast N7-methyltransferase complementation assays; however, the phenotypes of the corresponding mutant viruses differed remarkably. rMHV_nsp14-Y414A_ replicated to titers comparable to those of WT MHV in cell culture, rMHV_nsp14-N380A_ was severely impaired in virus replication and rapidly acquired a compensatory 380S substitution, and rMHV_nsp14-N416A_ did not replicate at all. These differences may reflect structural constraints that differ substantially dependent on whether coronavirus N7-MTase is expressed and assessed individually *in vitro* or whether it is embedded in the coronavirus multienzyme replicase-transcriptase complex (RTC). This complex may provide structural support and stability resulting from interactions between RTC components and N7-MTase cofactors, which compensate for the nsp14 mutations to differing extents. Accordingly, the residual N7-MTase activities of our mutants in the context of viral replication can be ranked as follows: rMHV_nsp14-Y414A_ > rMHV_nsp14-N380A_ > rMHV_nsp14-N416A_.

Based on the observed diverse phenotypes of the N7-MTase mutants, we draw a number of conclusions. First, dead mutant rMHV_nsp14-N416A_ may indeed encode an inactive N7-MTase, suggesting that this enzymatic activity is absolutely required for CoV replication. Second, mutants that may encode an N7-MTase with minimal residual activity, such as rMHV_nsp14-N380A_, can undergo rapid phenotypic reversion following a rapid genetic change, suggesting a strong selection for a sufficiently active N7-MTase and corroborating the critical role of N7-methylation of coronaviral mRNAs and/or for viral RNA replication. Notably, a similar observation has been made by Zhang and colleagues, who generated a West Nile virus mutant (family *Flaviviridae*) encoding an N7-MTase with only 1 to 2% residual activity. This mutant also underwent rapid phenotypic reversion (44). Third, our results provided further evidence for a critical role of nsp14 in MHV pathogenesis and innate immune evasion. This was demonstrated by a detailed phenotypic analysis of rMHV_nsp14-Y414A_, a virus that was viable and allowed us to experimentally assess the effects of partially defective N7-methylation on the innate immune response.

Based on the crystal structures of SARS-CoV and SARS-CoV-2 nsp14 (31, 32) and modeling of MHV nsp14, the side chain of MHV Y414 encompasses part of a pocket that stabilizes and positions the purine moiety of the guanosine of GpppN-RNA. Our results are most consistent with partial but not complete abrogation of N7-methylation by the Y414A replacement, since rMHV_nsp14-Y414A_ exhibited plaque sizes and growth kinetics in cell culture similar to those of WT rMHV (Fig. 1). Importantly, infection with rMHV_nsp14-Y414A_ resulted in upregulation of the expression of IFN-β and other innate immune mediators in BMDCs (Fig. 2) and in mice at early times p.i. (Fig. 6) (34). Infection with rMHV_nsp14-Y414A_ resulted in attenuated virulence after either i.h. or i.c. inoculation, demonstrating an important role for N7-methylation in infection of both brain and liver (Fig. 5). Further, viral spread to the liver after i.c. infection was impaired in rMHV_nsp14-Y414A_-infected mice, and this was reversed in infected *Ifnar*^−/−^ mice, reflecting either higher virus loads in the brains of *Ifnar*^−/−^ mice prior to spread or a robust and protective IFN-I-mediated immune response in the livers of rMHV-infected mice (Tables 1 and 2). Our *in vivo* study also implies that the relative high levels of IFNs produced at early stages of coronaviral infections are important in reducing pathogenesis and mortality. These results were supported by studies in which mice infected with rSARS-CoV-2_nsp14-Y420A_ but not those infected with rSARS-CoV-2 survived. In contrast, unchecked coronavirus replication may induce high levels of IFNs at later times after infection, causing a dysregulated acute inflammatory response, as occurs in many patients with severe SARS, MERS, and COVID-19 (Fig. 5 and 6) (45).

Assembly of a cap-1 structure at the 5′ end of viral mRNA is required for optimal translation and for evasion of the host immune response (5, 6, 46, 47). Formation of this cap in CoV-infected cells requires the sequential function of several enzymes, including RNA triphosphatase (TPase) (nsp13), N7-methyltransferase (nsp14), and 2′-O-methyltransferase (nsp16) (10, 47). 2′-O-methylation of viral RNA is critical to avoid its recognition by RNA sensors, such as MDA5 and RIG-I (6, 48). Thus, increased IFN-I responses elicited by rMHV_nsp14-Y414A_ may reflect the absence of 2′-O-methylation of the mutant RNA, since this methylation depends on N7-methylation, which was impaired in the nsp14 mutant (19). However, BMDC transfection with a panel of partially methylated MHV-derived RNA substrates revealed an independent role for N7-methylation in inhibiting IFN-β induction (Fig. 2f). In one study, RNA that was 2′-O- but not N7-methylated was produced *in vitro* and was shown to evade recognition by RIG-I, while RNA that was only N7-methylated exhibited modest RIG-I-evasive activity (48). Thus, N7-methylation prevents IFN-β induction through a pathway independent of facilitating 2′-O-methylation, which is critical for inhibiting IFN-β induction by cytoplasmic helicase sensors.

Of note, the cap is necessary for RNA translation (46). However, our results suggest that translation was not diminished by the absence of N7-methylation since we observed no differences in virus replication in cells lacking an IFN-I response. Further, while differences in capping might differentially affect genomic and subgenomic stability and translation, this has not been reported and should be investigated in future studies.

It is notable that 2′-O-methylation may precede N7-methylation in the capping of some viral mRNAs, such as those contained in rabies and measles viruses (46). Moreover, mRNAs from alphaviruses, such as chikungunya virus, and from orthohepeviruses, such as hepatitis E virus, are only N7-methylated (46). These results are consistent with the notion that N7-methylation is sufficient to impair IFN-I expression (49, 50). It is also possible that another protein or modification compensates for the lack of 2′-O-methylation in alphaviruses.

In summary, N7-methylation of the coronaviral RNA cap is important for evasion of the host immune response and maintaining its pathogenicity in at least two ways. First, N7-methylation is required for subsequent 2′-O-methylation. Second, N7-methylation, even in the absence of 2′-O-methylation, countered IFN-I induction. Therefore, N7-methylation is essential for the evasion of the host immune response and pathogenicity when viral RNA contains either 5′ N7-methylated cap-0 or N7/2′-O-methylated cap-1 structures, indicating that viral N7-methyltransferase is a useful target for the development of antiviral drugs and a useful component of live attenuated vaccines.

During the process of revision and resubmission, a paper characterizing N7-MTase structurally, biochemically, and virologically was published (51). That article complements our study.

## MATERIALS AND METHODS

### Cell culture and animals.

Murine L2 fibroblasts cells, 17Cl-1 cells, and HEK293T cells were maintained in Dulbecco′s modified Eagle medium (DMEM) supplemented with 10% fetal bovine serum (FBS) (Gibco), HEPES (10 mM), and 1% penicillin-streptomycin (Gibco). Sendai virus (SeV) and vesicular stomatitis virus expressing green fluorescent protein (VSV-GFP) were a kind gift from Hong-Bing Shu (Wuhan University). The recombinant SARS-CoV-2 used in these studies was passaged on Calu-3 cells. Calu-3 cells were grown in MEM (GIBCO) supplemented with 20% FBS. Vero E6 and Vero81 cells were grown in DMEM (GIBCO) supplemented with 10% FBS.

C57BL/6 mice were maintained at Wuhan University. *Ifnar^+/−^* mice were a kind gift from Bo Zhong (Wuhan University). For generation of *Ifnar*^−/−^ mice, male and female *Ifnar^+/−^* mice were crossed. To determine mouse genotypes, genomic DNA was assessed by PCR with the following primers specific for *Ifnar*: 5′-CGAGGCGAAGTGGTTAAAAG-3′ (common forward primer), 5′-ACGGATCAACCTCATTCCAC-3′ (wild-type reverse primer), and 5′-AATTCGCCAATGACAAGACG-3′ (mutant reverse primer). K18-hACE2 cells were generated as previously described (52) and provided by the Jackson Laboratories. All animal experiments were conducted in accordance with the regulations of Hubei Province Laboratory Animal Management and were approved by the Wuhan University Animal Experiment Ethics Committee or after approval by the University of Iowa Institutional Animal Care and Utilization Committee.

### Construction of plasmids.

The coding sequence for nonstructural protein 14 (nsp14) of MHV was amplified from infected cells, and the amplified fragment was inserted into the expression vector pET30a (Novagen). Mutant nsp14 fragments were generated by overlap PCR. To construct plasmids for functional screening in yeast, the amplified nsp14 fragments were cloned into the vector pMceK294A under the control of the yeast TP11 promoter as described previously (18). All clones and mutations were confirmed by DNA sequencing. All constructs encoded protein with an N-terminal 6×His tag.

### Expression and purification of recombinant proteins.

DH5a cells were transformed with PET30a plasmids, and protein was induced with 0.4 mM isopropyl-β-d-thiogalactopyranoside (IPTG) at 16°C for 12 to 16 h. nsp14 was purified with nickel-nitrilotriacetic acid (Ni-NTA) resin (GenScript) as described previously (19, 24).

### Yeast strain and assays of N7-MTase activity *in vivo*.

As described previously (18, 24), yeast strain YBS40 carries a deletion at the chromosomal Abd1 locus, encoding the yeast cap MTase, and its growth depends on the maintenance of plasmid p360-ABD (URA3) on agar medium containing 5-fluoroorotic acid (5-FOA). For analysis of MHV N7-MTase activity, yeast cells were transformed with expression plasmids carrying wild-type and mutant nsp14. Trp^+^ transformants were selected at 30°C on agar medium lacking tryptophan, and cells were streaked on agar medium containing 0.75 mg/ml of 5-FOA to counterselect URA3 plasmids at 30°C. Plates were incubated for up to 5 days, and the formation of FOA-resistant colonies indicated that the transformed mutants could replace or complement endogenous cap N7-methyltransferase.

### Biochemical assays for methyltransferase activity.

MTase functional assays were carried out in 30-μl reaction mixtures (40 mM Tris-HCl, pH 8.0), 2 mM MgCl_2_, 2 mM dithiothreitol (DTT), 40 units RNase inhibitor, 0.01 mM SAM) containing 1 μCi of *S*-adenosyl-[*methyl*-^3^H]methionine (67.3 Ci/mmol, 0.5 μCi/μl), 1 μg of mutant protein, and 3 μg of the GpppA-RNA substrate at 37°C for 2 h. The ^3^H-labeled product was purified using DEAE-Sephadex columns and quantitated by liquid scintillation. The GpppA-RNA (GpppAGAUUAGGUUUUUACCUACCCUG) substrate was prepared and purified as previously described (18).

### Preparation of uncapped/capped RNA and RNA transfection.

As described previously (19, 24), the 51-nt RNA (pppGN50, 5′-GUAUAAGAGUGAUUGGCGUCCGUACGUACCCUCUCAACUCUAAAACUCUUG-3′) of the 5′-UTR of the MHV-A59 genome was *in vitro* transcribed using a MEGAscript T7 transcription kit (Invitrogen). pppGN50 was capped with guanosine or N7-methyl guanosine (m7G) to synthesize GpppGN50 or m7GpppGN50, respectively, using a vaccinia virus capping system (NEB). m7GpppGN50 was further methylated into m7GpppGmN50 using RNA cap 2′-O-methyltransferase (NEB). The RNAs (50 nM) were transfected into BMDCs using Lipofectamine 2000 transfection reagent (Invitrogen).

### *In vitro* methylation of poly(A)-containing RNA.

Poly(A)-containing RNA was isolated from approximately 1 × 10^7^ mock-, rMHV-, or rMHV_nsp14-Y414A_-infected neuro2A cells at 24 hpi (multiplicity of infection [MOI], 1) with a Dynabeads mRNA purification kit (Invitrogen). The purified coronavirus nsp14 proteins were mixed at a final concentration of 200 nM with purified RNAs as the substrate. After incubation at 37°C for 1.5 h, the reaction was stopped by adding an equal volume of stop solution (0.2% SDS and 20 mM EDTA). The samples were purified using DEAE-Sephadex columns, and methylation of the RNA substrates was quantitated using a scintillation counter (Beckman Coulter LS 6500).

### Generation of recombinant coronaviruses.

Generation of rMHV and its mutants was performed as described previously (34). Recombinant vaccinia virus inf-1 (vMHV-inf-1) DNA, which contains a full-length MHV-A59 cDNA clone (GenBank accession no. NC001846) (53), has been used to generate full-length cDNA clones of rMHV, rMHV_nsp14-Y414A_, rMHV_nsp14-N380A_, rMHV_nsp14-N416A_, and rMHV_nsp14-D330A_.

rSARS-CoV-2 and mutant rSARS-CoV-2_nsp14Y420A_ were engineered using a Kan^r^-I-SceI marker cassette for dual (positive and negative) selection as previously described (54). pBAC-SARS-CoV-2 was kindly provided by Luis Enjuanes. Virus titers were measured as previously described (43). The sequences of nsp14 in rSARS-CoV-2 and rSARS-CoV-2_nsp14Y420A_ and of rescued virus were confirmed prior to use. The forward primer was TGTAGATTTGACACTAGAGTGCTATCTAACCTTAACTTGCCTGGTTGTGATGGTGGCAGTTTGgcTGTAAATAAACATGC, and the reverse primer was TAAATTAACAAAAGCACTTTTATCAAAAGCTGGTGTGTGGAATGCATGTTTATTTACAgcCAAACTGCCACCATC. (“gc” defines the site of mutation.)

### Virus growth and plaque assay.

17Cl-1 or L2 cells were infected at different MOIs as indicated in the figures. After 1 h at 37°C, the inoculum was removed and replaced with prewarmed medium. Samples were removed at the indicated time points, and titers were determined as follows. 17Cl-1 or L2 cells were grown in 60-mm dishes to 70 to 80% confluence and infected with 1 ml of media containing viruses at dilutions ranging from 10^−3^ to 10^−6^. After 1 h at 37°C, the inocula were removed and cells overlaid with 0.95% agar (Amresco). Plaques were picked at 24 to 36 hpi. For plaque staining, 3 ml of agar containing 0.02% neutral red (Sigma-Aldrich) was laid over cells. Six to 8 h later, stained plaques were counted.

SARS-CoV-2 was grown as previously described (43). Briefly, the recombinant SARS-CoV-2 used in these studies was passaged on Calu-3 cells, which were grown in DMEM (GIBCO) supplemented with 20% FBS.

### Preparation of BMDCs and cytokine detection.

Bone marrow cells were isolated from mouse tibia and femur. For the preparation of BMDCs, bone marrow-derived cells were cultured for 7 to 9 days in medium containing mouse granulocyte-macrophage colony-stimulating factor (GM-CSF; 20 ng/ml; Peprotech). The concentrations of IFN-β in culture supernatants and sera were measured using a mouse IFN-β enzyme-linked immunosorbent assay (ELISA) kit (BioLegend). To measure IFN-β secretion induced by rMHV and its mutants, L2 cells were infected with rMHV, rMHV_nsp14-Y414A_, or Sendai virus (positive control). The IFN-β concentration in the culture supernatants was measured by monitoring the inhibition of VSV-GFP replication in L2 cells.

### Mouse infection.

Three- to 4-week-old C57BL/6 mice or *Ifnar*^−/−^ mice were infected by intracranial or intrahepatic inoculation with rMHV or rMHV_nsp14-Y414A_ at the indicated MOIs. Mouse weight and mortality were monitored daily. Three infected mice were sacrificed at each indicated time point, and livers and brains were harvested or stored at −70°C for subsequent assays.

For SARS-CoV-2 infection, 6- to 8-week-old mice were intranasally infected with the indicated amount of SARS-CoV-2 in a total volume of 50 μl DMEM. Mouse weight and mortality were monitored daily, or lungs were collected at 3 and 10 dpi for histopathological analysis. All experiments with SARS-CoV-2 were performed in a biosafety level 3 (BSL3) laboratory at the University of Iowa.

Viral titers were determined by plaque assay. Sera were assayed for alanine transaminase (ALT) levels using a commercial kit (Nanjing Jiancheng Bioengineering Institute). For routine histology, the mouse livers or lungs were fixed in zinc formalin, embedded in paraffin, sectioned (approximately 4 μm each), and stained with hematoxylin and eosin (H&E).

### RNA isolation and qRT-PCR.

Total RNA was isolated from cells or liver using TRIzol reagent (Invitrogen). RNA was reverse transcribed to cDNA, which was used as the substrate for quantitative reverse transcription-PCR (qRT-PCR). GAPDH (glyceraldehyde-3-phoshpate dehydrogenase) mRNA served as an internal control. Transcript levels were quantified by comparative real-time PCR using the following gene-specific primers: MHV-RNA7 (sense, 5′-TATAAGAGTGATTGGCGTCC-3′; antisense, 5′-GAGTAATGGGGAACCACACT-3′), IFN-β (sense, 5′-AACCTCACCTACAGGGCGGACTTCA-3′; antisense, 5′-TCCCACGTCAATCTTTCCTCTTGCTTT-3′), and GAPDH (sense, 5′-CGACTTCAACAGCAACTCCCACTCTTCC-3′; antisense, 5′-TGGGTGGTCCAGGGTTTCTTACTCCTT-3′).

### RNA-seq pipeline.

Dendritic cells were infected at an MOI of 1 PFU/cell with rMHV-A59 or mutant virus. After 1 h at 37°C, the inocula were removed, and cells were covered with prewarmed medium. Samples were removed at 6 h. RNA was extracted using an RNAsimple total RNA kit (Tiangen DP419).

An RNA-seq library was constructed using an NEBNext RNA-seq preparation kit. RNA-seq libraries were sequenced using HiSeq X-10 with the 150-bp paired-end sequencing mode. Quality control of RNA-seq data was performed using Fatsqc, and low-quality bases and adaptor contamination were removed by Cutadapt. Data were then mapped to mouse reference genome mm10 by tophat2. For each read, we allowed a maximum 2-base mismatch. Cufflinks (55) was used to assemble transcripts and identify differentially expressed genes. Gene ontology analysis was performed using DAVID (https://david.ncifcrf.gov).

### siRNAs and transfection.

Small interfering RNAs (siRNAs) targeting *RIG-I* and *MDA5* were synthesized and transfected with Lipofectamine RNAiMAX transfection reagent according to the manufacturer′s manual. Twenty-four hours after transfection, cells were harvested or stimulated, followed by qRT-PCR assays.

The sequences of siRNAs are as follows: siRIG-I-1, 5′-GCAAGCATTCAGAGACTATATC-3′; siRIG-I-2, 5′-CGGACTTCGAACACGTTTAAA-3′; siMDA5-1, 5′-GCAAAGCAATACAACGACAATC-3′; and siMDA5-2, 5′-CCTACAAATCAACGACACGATC-3′.

### Statistical analysis.

Differences in mean values between groups were analyzed by analysis of variance (ANOVA) and Student’s *t* tests, and differences in survival were analyzed by log rank (Mantel-Cox) tests using GraphPad Prism 8. All results are expressed as means ± standard deviations (SD) and were corrected for multiple comparisons. A *P *of <0.05 was considered statistically significant (*, *P* ≤ 0.05; **, *P *≤ 0.01; ***, *P* ≤ 0.001).
