# Supplementary figures and images for: N7-Methylation of the Coronavirus RNA Cap Is Required for Maximal Virulence by Preventing Innate Immune Recognition
Source: mBio. 2022 Jan 25;13(1):e03662-21. doi: 10.1128/mbio.03662-21 (PMC8787479; doi:10.1128/mbio.03662-21)

Figure S1

(a)

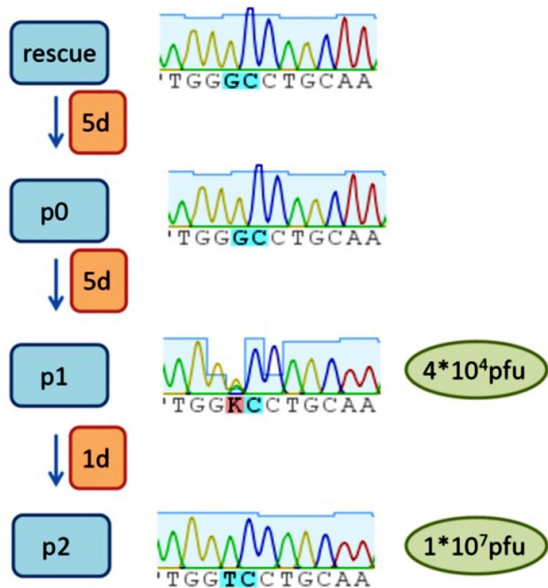

(b)

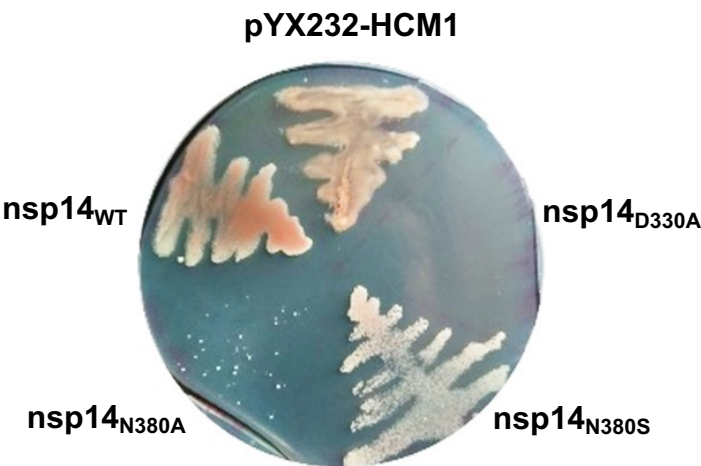

(c)

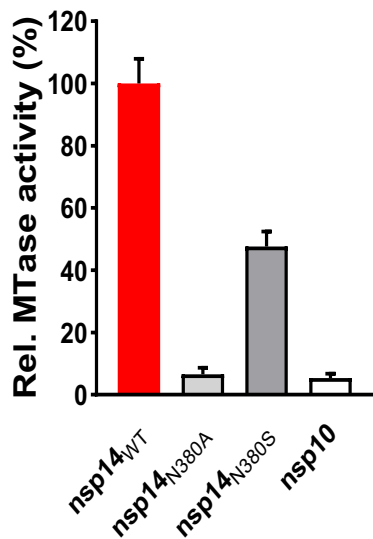

(d)

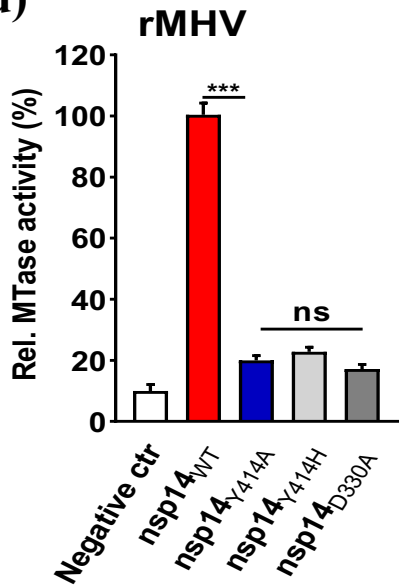

Supplement: FIG S1 [file mbio.03662-21-sf001.pdf]

Figure S2

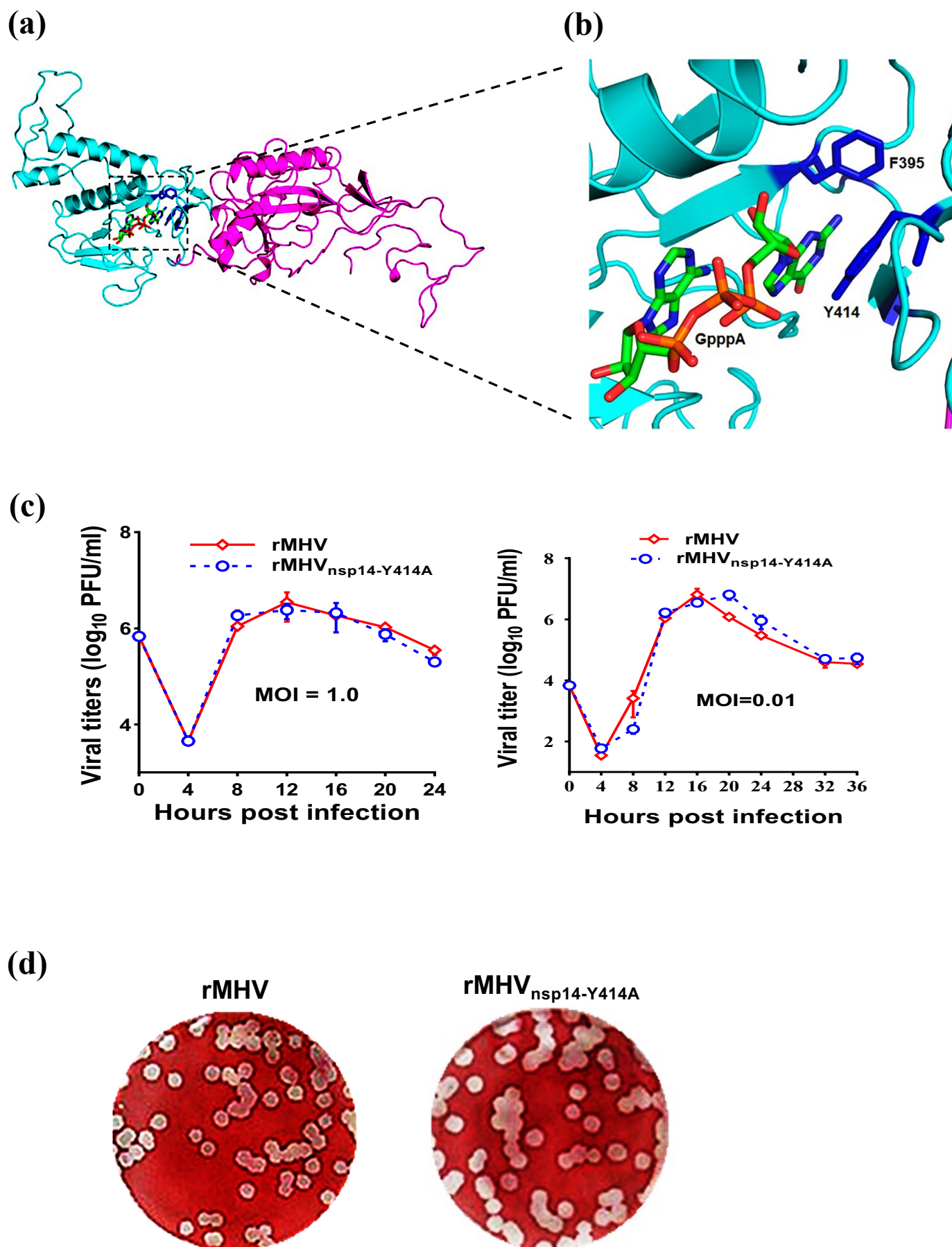

Supplement: FIG S2 [file mbio.03662-21-sf002.pdf]

**Figure S3**

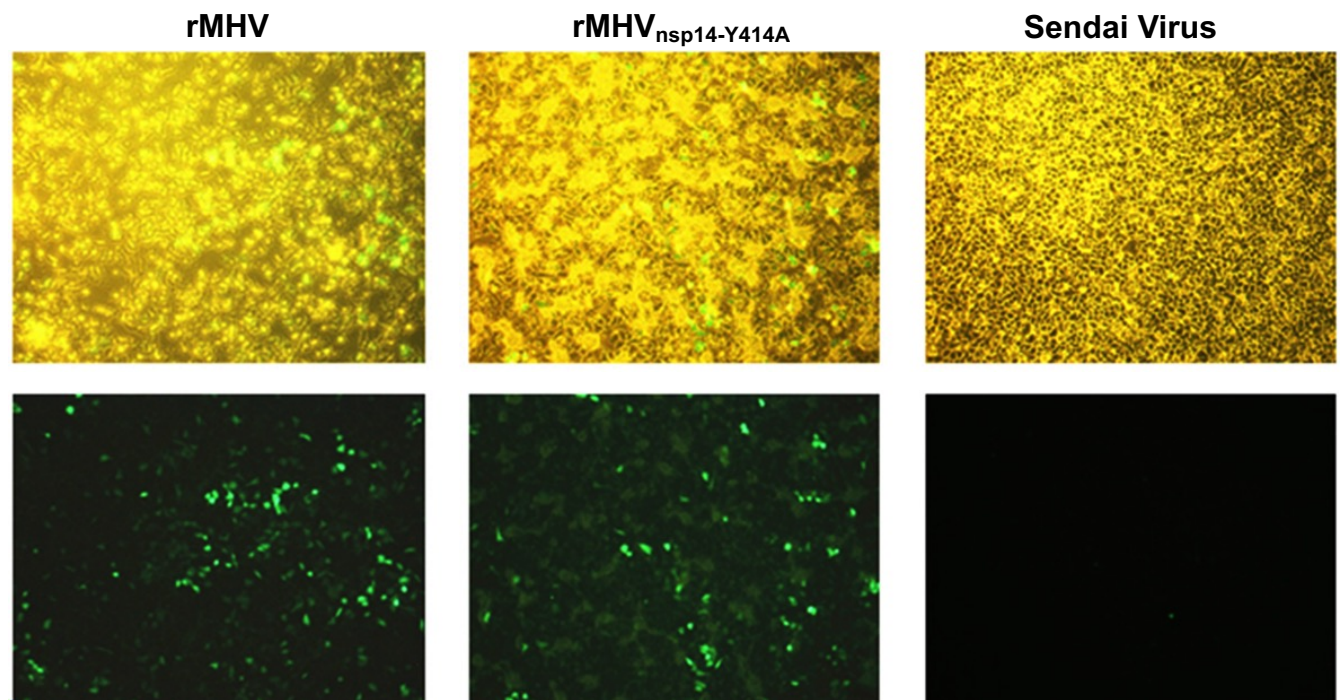

Supplement: FIG S3 [file mbio.03662-21-sf003.pdf]

Figure S4

(a)

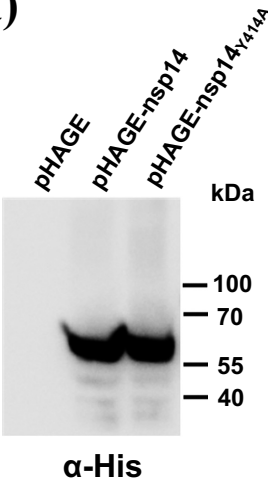

(b)

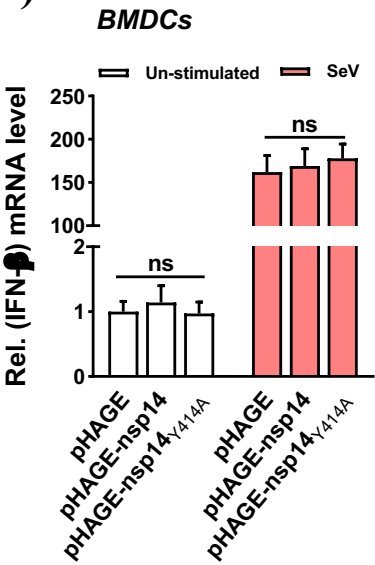

Supplement: FIG S4 [file mbio.03662-21-sf004.pdf]

**Figure S5**

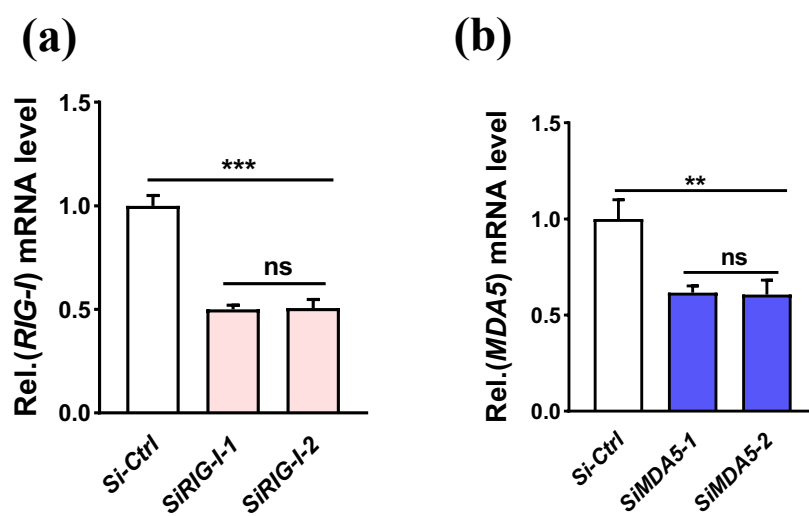

Supplement: FIG S5 [file mbio.03662-21-sf005.pdf]
